# Integrated Analysis of DNA Methylome and Transcriptome Reveals Epigenetic Regulation of Cold Tolerance in *Litopenaeus vannamei*

**DOI:** 10.3390/ijms241411573

**Published:** 2023-07-18

**Authors:** Weilin Zhu, Chunling Yang, Qingyun Liu, Min Peng, Qiangyong Li, Huanling Wang, Xiuli Chen, Bin Zhang, Pengfei Feng, Tiancong Chen, Digang Zeng, Yongzhen Zhao

**Affiliations:** 1 Key Lab of Freshwater Animal Breeding, Key Laboratory of Agricultural Animal Genetics, Breeding and Reproduction, Ministry of Education, College of Fishery Huazhong Agricultural University, Wuhan 430070, China; 2 Guangxi Key Laboratory of Aquatic Genetic Breeding and Healthy Aquaculture, Guangxi Academy of Fishery Sciences, Nanning 530021, China

**Keywords:** DNA methylome, transcriptome, cold tolerance, *Litopenaeus vannamei*

## Abstract

DNA methylation is an important epigenetic modification that has been shown to be associated with responses to non-biological stressors. However, there is currently no research on DNA methylation in response to environmental signals in shrimp. In this study, we conducted a comprehensive comparative analysis of DNA methylation profiles and differentially expressed genes between two strains of *Litopenaeus vannamei* with significantly different cold tolerance through whole genome bisulfite sequencing (WGBS) and transcriptome sequencing. Between Lv-C and Lv-T (constant temperature of 28 °C and low temperatures of 18 °C and 10 °C) under cytosine-guanine (CG) environments, 39,100 differentially methylated regions (DMRs) were identified, corresponding to 9302 DMR-related genes (DMRGs). The DMRs were mainly located in the gene body (exons and introns). Gene Ontology (GO) analysis showed that these DMRGs were significantly enriched in cell parts, catalytic activity, and metabolic processes. Kyoto Encyclopedia of Genes and Genomes (KEGG) analysis showed significant enrichment of these DMRGs in pathways such as proteasome (ko03050), oxidative phosphorylation (ko00190), mTOR signaling pathway (ko04150), fatty acid metabolism (ko01212), and fatty acid degradation (ko00071). The comprehensive results suggested that *L. vannamei* mainly regulates gene expression in response to low temperatures through hypermethylation or demethylation of some genes involved in thermogenesis, glycolysis, the autophagy pathway, the peroxisome, and drug metabolism pathways. These results provide important clues for studying DNA methylation patterns and identifying cold tolerance genes in shrimp.

## 1. Introduction

Epigenetic variations can be autonomously generated by environmental changes, exhibiting phenotypic plasticity for important ecological traits [1,2]. Epigenetic-induced phenotypic variation is a reversible change that can regulate the organism’s rapid response to environmental fluctuations, thereby enhancing its adaptive ability to environmental stress [3,4,5]. The regulatory mechanisms of epigenetics mainly include DNA methylation, histone modification, chromatin remodeling, and non-coding RNA [6]. DNA methylation is an important epigenetic modification that is widely present in eukaryotic organisms such as mammals, plants, and fungi [7,8]. DNA methylation refers to the covalent modification of methylated deoxyribonucleotides at the fifth carbon atom of the deoxythymidine ring, which is transferred by the DNA methyltransferase (DNMT) using S-adenylmethionine (SAM) as a methyl donor [9]. As one of the most important regulatory phenomena expressed in epigenetics, DNA methylation has important functions such as regulating gene expression, maintaining the stability of genetic material, and establishing epigenetic patterns. It also plays important roles in transcriptional regulation, transposon inactivation, genomic imprinting, and other cellular processes [10,11,12]. Many studies have shown that DNA methylation is closely related to environmental factors, including heat stress, diet, and light exposure [13,14], and plays a significant role in various environmental stresses [15]. Under environmental stress conditions, methyl groups can rapidly and reversibly modify DNA, avoiding unnecessary recombination of genes and allowing for a more rapid response to environmental changes [16].

Environmental stress can induce significant DNA methylation changes in invasive marine invertebrates over a very short time scale, and this response depends on the type, size, and duration of the stressor [17]. In fish, DNA methylation plays an important role in responding to environmental temperature stress, with 25% of differentially methylated regions (DMRs) in the three-spined stickleback (*Gasterosteus aculeatus*) being closely related to temperature regulation, with 50 being common DMRs [18]. Changes in DNA methylation levels throughout the genome during specific developmental stages of fish due to global warming-induced temperature changes can impact gene expression levels related to environmental stress response [19]. In zebrafish embryonic fibroblasts (ZF4), gene methylation is involved in various cold response biological processes such as the antioxidant system, cell apoptosis, development, chromatin modification, and immune system, was significantly affected by low-temperature stress, indicating that these processes respond to cold stress by regulating DNA methylation [20]. To resist low salinity stress, the liver of *Cynoglossus semilaevis* can regulate the expression of igf2 through methylation of an intron 1 CpG site [21]. In addition, changes in the methylation patterns induced by temperature stress can also regulate the sex of organisms. For example, in European perch (*Perca fluviatilis*), temperature-induced changes in methylation levels of the cyp19a promoter in the gonads can affect sex ratios [22]. Temperature can also affect DNA methylation patterns in the bipotential gonads of sea turtles (*Lepidochelys olivacea*), regulating the expression of Sox9, a gene associated with sex determination, and determining the development of the ovaries or testes [23]. Varriale et al. [24] found that DNA methylation is inversely proportional to body temperature, with the DNA methylation levels of polar fish being higher than those of tropical and temperate fish and the levels of methylation of tropical and temperate fish being higher than those of warm-blooded vertebrates, which is consistent with the results of Jabbari et al. [25]. Epigenetic studies in invertebrates have mainly focused on insects and mollusks. In the study of Diploptera punctata, it was found that overall DNA methylation variation was much lower at 28 °C compared to higher or lower temperatures [26]. The DNA methyltransferase (DNMT) activity in Epiblema scudderiana increases at low temperatures and decreases in high-temperature environments. The epigenetic regulation of genes and histones serves as the basis for the overwintering survival strategy of this insect [27]. A previous study conducted a comprehensive comparative analysis of the DNA methylation profiles of two strains of *Bombyx mori* silkworms that exhibited significant differences in heat and moisture tolerance and revealed that DNA methylation plays a crucial role in the response of silkworms to environmental stress [28]. Methylation-regulated genes have been identified in the genome of the bumblebee and have been shown to exhibit differential expression levels among queen bees, worker bees, and drones [29]. Environmental stressors, including temperature and salinity, can induce significant changes in DNA methylation in marine invertebrates, such as *Didemnum vexillum*, within a very short time scale. The nature, intensity, and duration of the stressor determine the extent of this response [17]. DNA methylation plays a potential functional role in oyster sex determination, with male gonads showing higher levels of DNA methylation and more genes with high methylation [30]. Whole-genome bisulfite sequencing (WGBS) was performed on two reef-building coral species with different environmental sensitivities. It was found that *Pocillopora acuta*, which is more sensitive to methylation, exhibited a methylation rate of 2.9%, while Montipora capitata had a methylation rate of 11.4%. The majority of CpG methylation in both species occurred in gene bodies and flanking regions [31]. *Daphnia magna* exhibits an impact on the whole-genome methylation profile under heat-restricted conditions [32].

The Pacific white shrimp (*Litopenaeus vannamei*), also known as the white shrimp or the whiteleg shrimp, is one of the three most commonly farmed shrimp species in the world [33]. Low temperatures are one of the main environmental factors that affect the growth and survival of *L. vannamei*. The suitable growth temperature for *L. vannamei* is 25–35 °C, and its tolerance to low temperatures is poor. It stops feeding when the temperature is below 18 °C [34], which limits the season and region for shrimp farming and affects the economic benefits of shrimp farming. Given that low-temperature environments may affect the DNA methylation levels of aquatic organisms, there are currently no reports on DNA methylation in *L. vannamei* regarding temperature stress. Therefore, this study constructed DNA methylation maps for two *L. vannamei* strains (cold-tolerant and normal strains) from sodium bisulfite sequencing and compared the distribution of CG, CHG, and CHH methylation levels under low-temperature stress. In addition, we performed transcriptome sequencing, focusing on the overall genome DNA methylation pattern and conducting a whole-genome association analysis of DNA methylation and gene expression. The aim of this study is to explore the shrimp methylation genome pattern and changes related to low-temperature stress, as well as identify key genes involved in cold tolerance based on methylation patterns.

## 2. Results

### 2.1. Genome-Wide DNA Methylation Patterns in Response to Cold Stress

To understand the response patterns of genomic DNA methylation characteristics to low-temperature effectiveness, we detected the overall DNA methylation levels of representative low-temperature-tolerant Lv-T and common strain Lv-C liver and pancreas tissues through whole-genome bisulfite sequencing. Overall, we obtained 43,817,521,500 to 64,194,630,150 raw reads from 18 DNA library samples using bisulfite sequencing (Table 1). We filtered out raw reads with an N content greater than 10% and low-quality reads (bases with a quality value Q < 20 accounted for more than 40% of the entire read), resulting in 270,324,896 to 414,526,126 clean reads. Among them, an average of 72.88% was mapped to the shrimp genome, with a sequencing depth of 17.01–27.1 and up to 50× sequencing depth. The detected cytosine numbers reached saturation (Figure S1), and bisulfite conversion efficiency ranged from 98.72% to 99.36% per sample.

The DNA sample used for analysis is a multicellular sample, so the level of methylation of C bases is a numerical value within the range of 0% to 100%, which is equal to the sum of the methylation levels of C bases with effective coverage divided by the total number of bases with effective coverage. The overall characteristic of the genome methylation map is reflected in the average methylation level of the whole genome (C bases are divided into three types based on their sequence characteristics: CG, CHG, and CHH). Based on methylation sequencing, the average methylation rate of the whole genome observed at the C position was 1.2%, 4.58%, 0.51%, and 0.56% for CG, CHG, and CHH, respectively (Supplementary Table S1).

**Figure 1:**
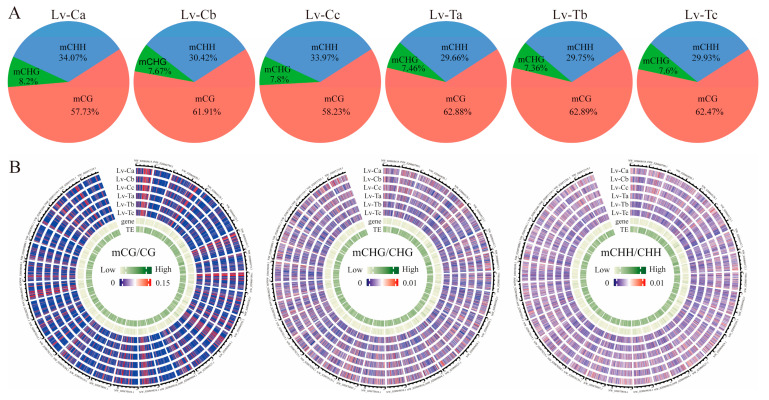
Characteristics of DNA methylation in *Litopenaeus vannamei*. (**A**) Relative proportions of mCs in three sequence contexts (CG, CHG, and CHH). (**B**) A circos plot of gene and transposon density and mCG, mCHG, and mCHH location in soybean.

The Lv-T28 (cold-tolerant strain at 28 °C) genome showed 62.88% mCG (mCG/CG), 7.64% mCHG (mCHG/CHG), and 29.66% mCHH (mCHH/CHH), indicating the percentage of methylation levels in the *L. vannamei* genome. Correspondingly, Lv-C28 (the normal strain at 28 °C) presented 57.73%, 8.2%, and 34.07% in CG, CHG, and CHH contexts, respectively. We can see the difference in methylation levels between cold-tolerant and normal strains. Under low-temperature stress, the Lv-T10 (cold-tolerant strain at 10 °C) genome presented 62.47% mCG (mCG/CG), 7.6% mCHG (mCHG/CHG), and 29.93% mCHH (mCHH/CHH), which showed the percentage of methylation levels in the *L. vannamei* genome. Correspondingly, Lv-C10 (normal strain at 10 °C) presented 58.23%, 7.8%, and 33.97% in CG, CHG, and CHH contexts, respectively. While investigating the distributions of mCs in three sequence contexts, we observed that methylcytosine was most common at the CG sites (57.73–62.89%) and occurred less frequently in CHH and CHG sequences (29.75–34.07% and 7.38–8.2%, respectively). Under low-temperature stress, the proportion of mCG in cold-tolerant strains slightly decreased, while the proportions of mCHG and mCHH slightly increased. The opposite trend was observed in normal strains, with the proportion of mCG increasing and the proportions of mCHG and mCHH decreasing (Figure 1A). The whole-genome DNA methylation map showed that high methylation occurred in regions rich in transposable elements (TEs), while regions rich in genes showed relatively low methylation in *L. vannamei* (Figure 1B).

In all mCGs and mCHH, the DNA sequence logo of methylated cytosine showed a consistent bias towards the sequence under low-temperature stress, regardless of whether it was a cold-tolerant or normal strain, while the mCHG logo showed differences (Figure S2). Different distribution types of C bases (CG, CHG, and CHH) have different levels of methylation among different species and even different cell types of the same species. Over 90% of the C bases in the liver and pancreas methylation types of shrimp were distributed in the methylation level range of 0–5% for CG, CHG, and CHH (Figure S3). There is a tendency for highly methylated CG sites ranging between 90 and 100% and CHG and CHH to have low methylation of 20–30% (Figure S4), reflecting the DNA methylation characteristics of the species’ liver and pancreas.

### 2.2. DNA Methylation Patterns in Gene and TE Regions

**Figure 2:**
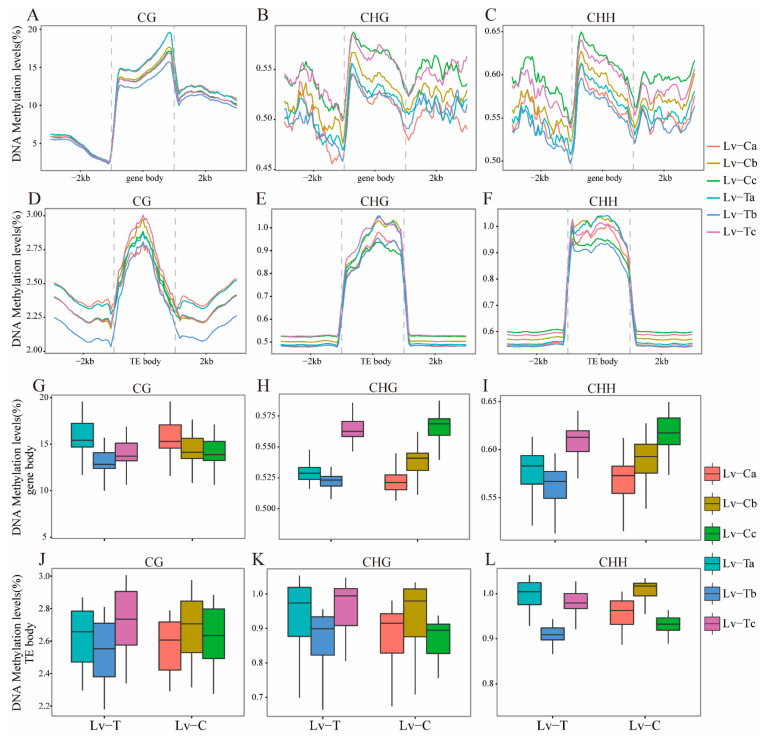
Comparison of DNA methylation patterns of genes and transposable element regions between the cold-tolerant family and the common family. (**A**–**C**) Metaplot of CG (**A**), CHG (**B**), and CHH (**C**) methylation of genes between cold-tolerant families and common family bases across the cold stress course. (**D**–**F**) Metaplot of CG (**D**), CHG (**E**), and CHH (**F**) methylation of transposable elements (TE) between cold-tolerant families and common family bases across the cold stress course. (**G**–**I**) Boxplot of CG (**G**), CHG (**H**), and CHH (**I**) methylation of gene bodies between cold-tolerant families and common family bases across the cold stress course. (**J**–**L**) Boxplot of CG (**J**), CHG (**K**), and CHH (**L**) methylation of transposable element body (TE body) between cold-tolerant families and common family bases across the cold stress course.

To explore the methylation patterns of different genomic structures, we examined the DNA methylation profiles of both gene and TE regions. We found that the cold-tolerant and normal strains had similar trends in CG, CHG, and CHH methylation under low-temperature stress, and this phenomenon was consistent in both gene and TE regions. The methylation level was relatively high in the gene body and TE body regions and low in the flanking regions for both CG, CHG, and CHH in the gene and TE regions (Figure 2A–F). In particular, TE body regions showed significantly higher methylation levels than the flanking regions (Figure 2D–F). In gene regions, CHG and CHH methylation levels increased at 10 °C after low-temperature stress, regardless of whether it was a cold-tolerant or normal strain (Figure 2B,C). In TE regions, the CG methylation level of the TE body region of the cold-tolerant strain was the highest at 10 °C under low-temperature stress, while the flanking regions showed a high methylation rate at normal temperature (Figure 2D). However, in CHG and CHH regions, the flanking regions showed a high methylation rate at 10 °C after low-temperature stress, regardless of whether it was a cold-tolerant or normal strain (Figure 2E,F).

To further investigate the temperature-induced changes in DNA methylation levels in gene and TE regions, we focused on the gene and TE body regions rather than the flanking regions. Based on DNA methylation data under low-temperature stress, we compared the DNA methylation levels of gene and TE body regions in cold-tolerant and normal strains of liver and pancreatic tissues at different temperature gradients. In the cold-tolerant strain (Lv-T), changes in CG, CHG, and CHH methylation with decreasing temperature and increasing temperature were observed in both gene body and TE body regions (Figure 2G–L). In contrast, in the normal strain, the CG methylation level in the gene body region decreased with decreasing temperature (Figure 2G), while the CHG and CHH methylation levels increased (Figure 2H,I). The trend of CG, CHG, and CHH methylation levels in the TE body region showed an initial increase followed by a decrease in both cold-tolerant and normal strains (Figure 2J–L). Overall, the CG, CHG, and CHH methylation levels in the gene body and TE body regions at normal temperature (28 °C) were higher in the cold-tolerant strain than in the normal strain. The CHG and CHH methylation levels at 10 °C after low-temperature stress were significantly higher than those at normal temperature (28 °C) in the gene body region of all strains. In the TE body region, the trend of CG, CHG, and CHH methylation levels was opposite in the cold-tolerant and normal strains, and the CG, CHG, and CHH methylation levels of the cold-tolerant strain at 10 °C under low-temperature stress were higher than those of the normal strain. In conclusion, the CG, CHG, and CHH methylation levels of cold-tolerant and normal strains showed different changes under low-temperature stress, which played an important role in adaptation to low-temperature stress.

### 2.3. Association between DNA Methylation and Gene Expression

**Figure 3:**
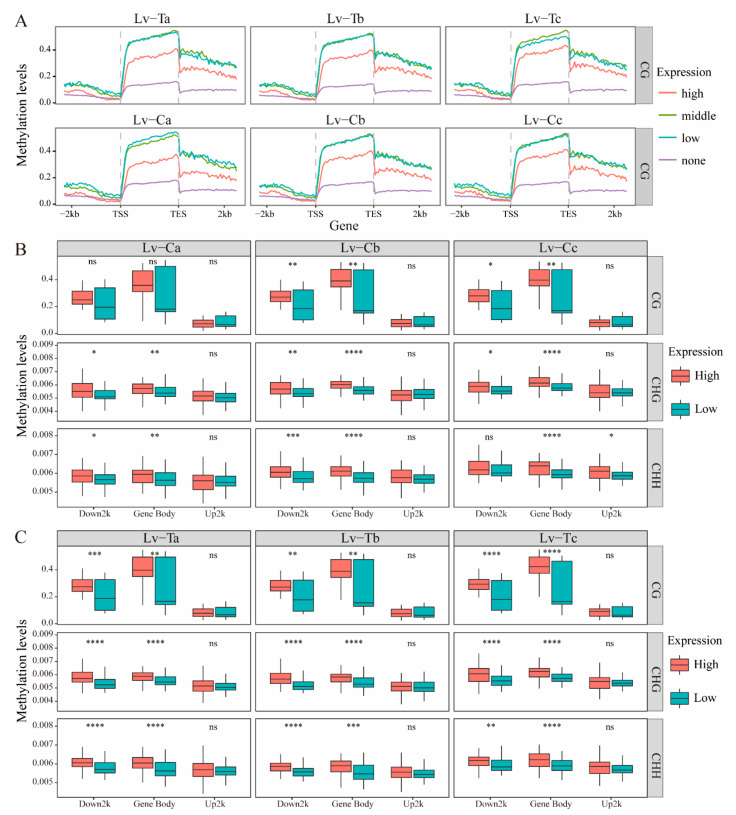
Methylation at various genic regions is differentially associated with gene expression. (**A**) Comparison of gene expression and methylation levels for mCG, mCHG, and mCHH sites and for each genic region: upstream 2 kb regions (Up2k), gene body, and downstream 2 kb regions (Down2K). On the basis of the expression of the upper and lower quantiles, the genes were divided into four groups: none (FPKM < 1); low (1 < FPKM < lower quantile); medium (lower quantile < FPKM < upper quantile); high (FPKM > upper quantile). The default selection is FPKM = 1, as the threshold gene is expressed. (**B**) and (**C**), the box plots of cold-resistant and ordinary families, respectively. The box plot of gene expression and methylation levels for mCG, mCHG, and mCHH sites and for each genic region: upstream 2 kb regions (Up 2k), gene body, and downstream 2 kb regions (Down 2k). Low expression (Low) included none (FPKM < 1) and low (1 < FPKM < lower quantile); High expression (High) included medium (lower quantile < FPKM < upper quantile) and high (FPKM > upper quantile). Asterisks indicate the statistical significance of the indicated differences (ns: not significant; *: *p* < 0.05; **: *p* < 0.01; ***: *p* < 0.001; ****: *p* < 0.0001; Mann–Whitney U test).

DNA methylation in different genomic regions varies and is associated with gene expression. In order to confirm the relationship between gene expression and DNA methylation, WGBS was used to measure DNA methylation using the same samples as those used for gene expression analysis. The DNA methylation profile of the entire gene region was examined. The DNA methylation level was significantly elevated in the upstream 2 kb and downstream 2 kb flanking sequences and was consistently away from the transcription start site (TSS) and termination site under all conditions. The regions between genes and upstream and downstream of the promoter showed a sharp decrease in methylation levels. In order to investigate the regulation of DNA methylation on gene expression in cold-resistant and normal family liver and pancreatic tissues, we preliminary tested the CG, CHG, and CHH methylation levels of all expressed genes in liver and pancreatic tissues. They were divided into four levels according to gene expression: none (FPKM < 1), low (1 < FPKM < lower quantile), medium (lower quantile < FPKM < upper quantile), and high (FPKM > upper quantile) (Figure 3A). The results showed that the CG methylation level in the middle and low expression groups was the highest among all temperature treatment groups. The CG methylation level in the high expression group was in the middle range, and the CG methylation level in the non-expression group was the lowest (Figure 3A). However, there was no apparent pattern in CHG or CHH in this study. In CG, CHG, and CHH methylation, methylation levels were positively correlated with gene expression in upstream, gene body, and downstream sequences, regardless of cold-resistant or normal families (Figure 3B,C). Figure S5 shows a positive correlation between the methylation in promoter and gene body regions and gene expression, as higher levels of promoter methylation showed higher levels of expression. In addition, Spearman correlation analysis was conducted between methylation and gene expression levels. As shown in Figure S6, the overall correlation for Rho was low regardless of methylation type. However, for CG methylation, Rho in the gene body region could reach 0.49. This result suggests that the methylation level in the gene body region and a part of the downstream 2 kb region has a relatively high correlation with its expression level. In all temperature gradients of the cold-resistant family, there were extremely significant differences in CG, CHG, and CHH methylation levels in the upstream 2 kb region and gene body, but not in the downstream 2 kb region (Figure 3B). In the normal family, the 18 °C treatment group (Lv-Cb) was similar to the cold-resistant family (Figure 3C); however, there was no significant difference in CG methylation in the room temperature treatment group (Lv-Ca), and the CHG and CHH upstream 2 kb methylation levels showed significant differences, while the gene body methylation level showed extremely significant differences and the downstream 2 kb region showed no significant differences. In the low-temperature 10 ℃ treatment group (Lv-Cc), CG and CHG upstream 2 kb methylation levels showed significant differences, while the gene body methylation level showed extremely significant differences and the downstream 2 kb region showed no significant differences. The methylation level in the CHH upstream and downstream 2 kb regions was opposite to that of CG and CHG, and the gene body methylation level showed extremely significant differences (Figure 3C).

### 2.4. Identification of DMR and DMR-Associated Genes in Response to Cold Stress

**Figure 4:**
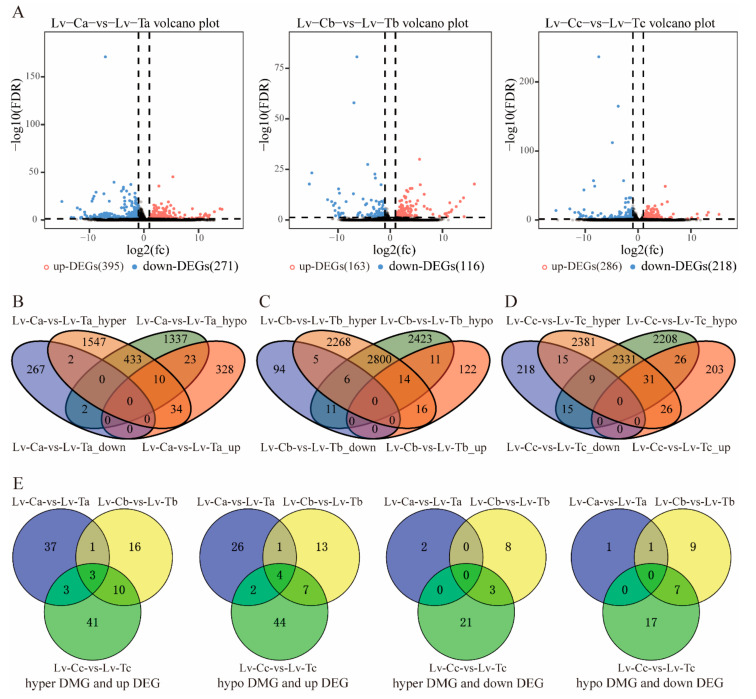
Changes in methylation affect gene transcription. (**A**) Differential expression genes (DEGs) of Lv-C vs. Lv-T respond to low-temperature stress. Each dot represents a gene, with red dots representing upregulated genes, blue dots representing downregulated genes, and black dots representing genes with no differential expression. The X-axis represents the log2 value of fold change, and the Y-axis represents the log10 value of false discovery rate (FDR). (**B**–**E**) are Venn diagrams of differentially methylated genes (DMRGs) and DEGs for each comparison group.

We used methylkit [31] (V1.4.1) software [35] for differential DNA methylation analysis. We first filtered out low-depth sequencing sites, requiring a minimum sequencing depth of at least four for C sites used in subsequent differential methylation analysis. We scanned the entire genome using a 200 bp window to calculate the average DNA methylation level of each type of C within each window and then compared the methylation levels of each sample in each window. We performed differential methylation analysis for all C, CG, CHG, and CHH sites using different filtering criteria. Cluster analysis also revealed widespread methylation group changes under cold stress (Figure S7). Among these DMRs, the proportion of CG was higher than that of CHG and CHH, consistent with most species’ research results [36,37]. The number of DMRs in the cold-tolerant group for CG, CHG, and CHH was higher than that in the normal temperature group, and the DMRs in the cold-tolerant group were higher than those in the common group (Figure S8). Based on the association between DMRs’ location and protein-coding genes and the 2 kb upstream and downstream regions, some overlaps were found between DMRs and genes (Figure 4B–D). Among these genes corresponding to these DMRs (Supplementary Table S2), it was found that *PSMD13*, *RPM26*, *PCIF1*, *HAT1*, and *ACAT2* had no change in methylation rate in the Lv-Ca vs. Lv-Ta comparison group but were upregulated in both the Lv-Cb vs. Lv-Tb and Lv-Cc vs. Lv-Tc groups after cold treatment. *COX6C*, *Scsalpha*, *SMAP1L*, *SYNRG*, *dcakd*, *Start1*, *PCIF1*, *Aldh2*, and *GSK3B* were downregulated in the Lv-Cb vs. Lv-Tb and Lv-Cc vs. Lv-Tc groups. *Dnase2*, *Actr1a*, *mms19*, *POLD3*, *PMT-2*, *USP47*, *NPEPL1*, *Rmnd1*, *Bbs5*, *Cpsf1*, *KdelR*, *ZNF540*, and *BTBD7* were upregulated in all three comparison groups (Lv-Ca vs. Lv-Ta, Lv-Cb vs. Lv-Tb, and Lv-Cc vs. Lv-Tc), while *ddx20*, *Trabd*, *Kmt2e*, *UGT2B31*, and *SraP* were downregulated in all three comparison groups. These genes are related to regulating energy metabolism, lipid metabolism, carbohydrate metabolism, and amino acid metabolism, indicating that these genes may be involved in methylation regulation under low temperature conditions.

**Figure 5:**
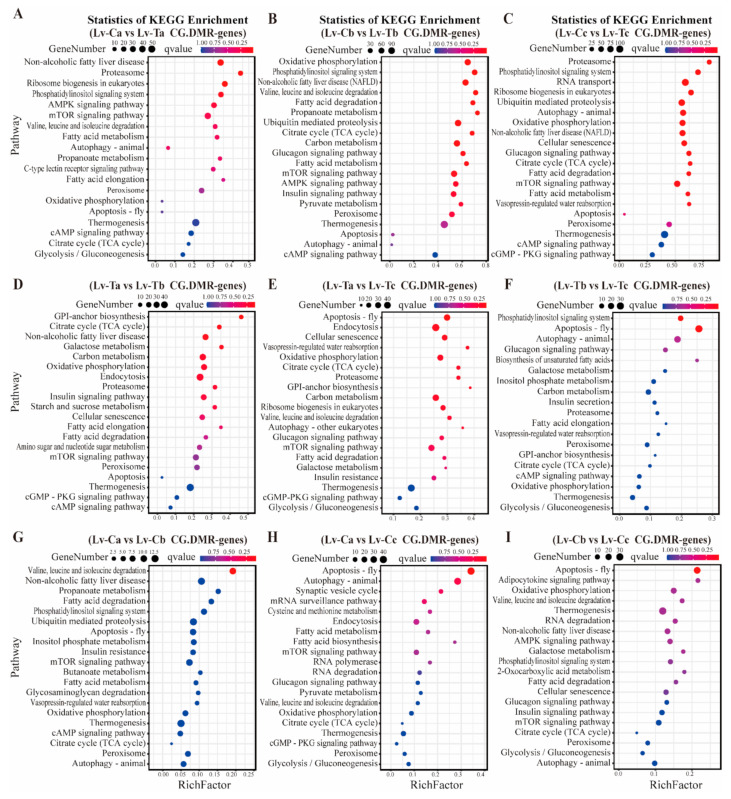
KEGG pathway enrichment analysis performed on differentially methylated genes related to differential methylation regions (DMRs) between cold-resistant and normal shrimp strains under low temperature stress. The size of the circle represents gene numbers, and the color represents the q-value. (**A**–**I**) represent different grouping comparisons.

Furthermore, we used KEGG (Kyoto Encyclopedia of Genes and Genomes) pathway enrichment analysis to identify pathways affected by low temperature treatment in both genotypes. The differentially enriched DMR genes in the Lv-T vs. Lv-C comparison were enriched in non-alcoholic fatty liver disease (ko04932), the proteasome (ko03050), oxidative phosphorylation (ko00190), and the mTOR signaling pathway (ko04150). After cold treatment at 18 °C and 10 °C, the significantly differently enriched DMR genes in the cold-tolerant group were enriched in fatty acid degradation (ko00071), citrate cycle (TCA cycle) (ko00020), and autophagy-animal (ko04140) pathways (Figure 5A–C), indicating that these pathways play an important role in corresponding low temperature stress. In addition, the significantly differently enriched DMR genes in the cold-tolerant group after cold treatment were also enriched in the glycosylphosphatidylinositol (GPI)-anchor biosynthesis (ko00563), citrate cycle (TCA cycle) (ko00020), carbon metabolism (ko01200), oxidative phosphorylation (ko00190), endocytosis (ko04144), proteasome (ko03050), and cellular senescence (ko04218) pathways (Figure 5D–I). Moreover, significant enrichment was found in apoptosis-fly (ko04214) and autophagy-animal (ko04140) under cold stress (Figure 5E,F,H,I), suggesting that these are important pathways related to cold tolerance.

### 2.5. Conjoint Analysis of Methylome and Transcriptome Alterations under Low Temperature Stress

To explore the gene expression changes that accompany widespread methylation changes in response to low temperature stress, RNA-seq analysis was performed on the same tissue under low temperature conditions. In the comparison of Lv-Ca vs. Lv-Ta, Lv-Cb vs. Lv-Tb, and Lv-Cc vs. Lv-Tc groups, a total of 844 upregulated DEGs and 605 downregulated DEGs were identified (Figure 4A). Between Lv-C vs. Lv-T, 24 differentially methylated regions (DMRs) associated with upregulated transcriptional genes and 11 differentially methylated regions associated with downregulated transcriptional genes were identified (Table 2; Figure 4B–E). Therefore, we found that one gene (*Rpn2*) was upregulated in transcriptional expression due to hypermethylation (Hyper), and four genes (*Hsd17b4*, *Tomm22*, *SdhD*, and *RAI14*) were downregulated due to hypomethylation (Hypo) and were downregulated at all three temperature gradient comparison groups. Under low-temperature treatment, *eIF2alpha*, *RPL6*, *SdhD*, *cct3*, *smim8*, *AK3*, *iolG*, *Riok2*, and *SdhD* were hypermethylated or demethylated (Hyper/Hypo) and downregulated in transcriptional expression, while *SAT2*, *mon2*, *GUSB*, *AK3*, *LMAN1*, *VPS11*, *dhtkd1*, *NDUFS3*, *SdhD*, and *Sin3a* were upregulated. Similarly, 26 differentially methylated regions associated with upregulated transcriptional genes and 37 differentially methylated regions associated with downregulated transcriptional genes were identified within the Lv-T lineage (Supplementary Table S3; Figure S9C,D), while there were twenty-two and eight in the Lv-C lineage, respectively (Supplementary Table S4; Figure S9A,B). These genes show changes in methylation levels as temperature changes, regulating the transcriptional expression level of genes, indicating that these genes may be involved in methylated regulation under low temperature conditions and are important genes related to cold tolerance.

## 3. Discussion

DNA methylation is the most well-known and extensively studied epigenetic mechanism involved in regulating gene expression [38]. DNA methylation is considered an important epigenetic marker, and recent studies have shown that DNA methylation is associated with regulating gene expression in response to non-biological stressors in many species, such as plants, fungi, and mammals [13,39,40,41]. Currently, the cytosine methylation level in most insects is about 0–1% [28,42], while it is 3–10% in mammals and birds, 10% in fish and amphibians, and up to 50% in some plants [43]. In this study, the average rate of C-site genome-wide methylation in shrimp was 1.2%, higher than that in silkworms and lower than that in fish and amphibians [38], indicating that different *L. vannamei* strains or environments may lead to different levels of DNA methylation. According to our mapping results, an average of 69.60% of cytosines were methylated in CpG sites. In the CHH and CHG contexts, only 0.51% and 0.56% of cytosines were methylated, respectively (Supplementary Table S1), which is consistent with findings in vertebrates [44].

This study investigated for the first time the DNA methylation patterns of two *L. vannamei* strains under low-temperature treatment and conducted a systematic analysis of DMRs to determine the important differentially methylated genes that may affect the development of the shrimp’s response to cold stress. Whole-genome bisulfite sequencing (WGBS) at single-base resolution showed that the methylation levels were 61.02% mCG (mCG/CG), 31.3% mCHG (mCHG/CHG), and 7.68% mCHH (mCHH/CHH) on average for the two strains. The methylation level of mCG in the cold-resistant strain (62.75% mCG/CG) was higher than that in the normal strain (52.29% mCG/CG), while the methylation levels of mCHG (29.78% mCHG/CHG) and mCHH (7.47% mCHH/CHH) were lower than those in the normal strain (32.82% and 7.89%, respectively). This indicates that different *L. vannamei* strains or environments may lead to different levels of DNA methylation.

Studies have shown that DNA methylation varies in different genomic regions and is associated with gene expression [45,46,47]. In this study, the highest levels of CG methylation were found in genes with middle and low expression levels, while the CG methylation levels of highly expressed genes were at intermediate levels, and the CG methylation levels of genes with no expression were the lowest (Figure 3A). However, there were no obvious patterns in CHG or CHH. Our research on *L. vannamei* showed that there was a positive correlation between methylation levels and gene expression in upstream, gene body, and downstream sequences in CG, CHG, and CHH methylation, consistent with previous studies on humans and bovines [48,49]. It has been reported that DNA methylation can inhibit gene expression [50], but recent genomic methylation studies have shown that the correlation between DNA methylation and transcription is different from what was initially thought. For example, recent methylome analyses of rice [51] and apple [52] have shown that gene body methylation is generally positively correlated with gene expression. These results suggest a more complex regulatory relationship between DNA methylation and gene expression, which seems to depend on the type, region, and degree of methylation, as well as the species. Our research on *L. vannamei* indicated a positive correlation between methylation levels and gene expression at upstream, genic, and downstream CG, CHG, and CHH methylation sites. This finding was consistent with studies conducted on bumblebees [53]. Recent studies on genome-wide DNA methylation have shown a positive correlation between DNA methylation and transcription. For example, higher levels of CpG methylation in bumblebees are associated with higher levels of gene expression [53]. Similar trends have also been observed in other social insects [7,54,55,56]. High levels of methylation in highly expressed genes are believed to play a role in the epigenetic regulation of caretaker genes in certain holometabolous insects [57,58,59]. High levels of genomic DNA methylation are also present in highly expressed genes in plants, such as rice and apples [60]. These studies suggest that there is a close regulatory relationship between DNA methylation and gene expression, which appears to depend on the type, region, and extent of methylation as well as the species involved.

The upstream 2 kb regions and gene body methylation levels of CG, CHG, and CHH were different in high and low expressed genes in the cold-resistant strain, especially the significantly different CG methylation levels under normal temperature, whereas there was no difference in the normal strain. Under cold stress, the differences in CHH methylation levels in high- and low-expressed genes in the cold-resistant strain appeared in the upstream 2 kb region, while in the normal strain, they appeared in the downstream 2 kb region. This indicates that the CG and CHH methylation patterns of high- and low-expressed genes play an important role in the adaptive response of shrimp to cold (Figure 3). In the functional regions of CG, CHG, and CHH sites, we found that methylation levels were highest in exon regions and peaked in gene body regions near the TSS, suggesting that DNA methylation is more likely to occur in the exon regions of genes in response to low-temperature stimulation in Pacific white shrimp, which is consistent with the results of silk moth (Bombyx mori) research [28].

Changes in DNA methylation under various stress conditions are usually associated with the regulation of gene expression. Therefore, in our study, we proposed and discussed whether and how DNA methylation is related to gene expression under low-temperature conditions. In this study, we identified 39,100 DMRs between the cold-resistant and normal strains, corresponding to 9302 DMRGs, and GO analysis showed that these DMRGs were most significantly enriched in the cell part, catalytic activity, and metabolic processes (Figure S10). KEGG analysis showed that these DMRGs were significantly enriched in the proteasome (ko03050), oxidative phosphorylation (ko00190), mTOR signaling pathway (ko04150), fatty acid metabolism (ko01212), and fatty acid degradation (ko00071), consistent with previous research [61], which provides important clues for studying DNA methylation patterns and identifying cold-resistant genes.

In addition, we also conducted a correlation analysis of DMRGs and DEGs to identify key genes related to cold resistance. We identified 24 upregulated genes and 11 downregulated genes that were differentially methylated in DMRs (Table 2; Figure 4E). We found that one gene (*Rpn2*) had hypermethylated expression upregulation, and four genes (*Hsd17b4*, *Tomm22*, *SdhD*, and *RAI14*) had hypomethylated expression downregulation. *Rpn2* is a homolog of the 26S proteasome non-ATPase regulatory subunit and is essential for embryonic, larval, and germline development [48]. *RAI14* is an ankyrin-corbin-like protein and a novel actin cytoskeleton-associated protein [62]. *RAI14* is an ankycorbin-like protein and a novel actin cytoskeleton-associated protein [63]. The Tomm mechanism is a molecular switch for PINK1 and PARK2/parkin-dependent mitochondrial clearance [64]. In addition, the methylation and gene expression of *CACNA1S*, *ATP8B2*, *TTC39B*, *NCX1*, *CCT3*, *RPL6*, and *Grx3* changed under cold stress. CACNA1S and NCX1 have low-temperature response and cold conduction functions [51,52], *ATP8B2* regulates plasmid homeostasis and plays a role in intracellular signal transduction [65], *TTC39B* is a member of the tetratricopeptide repeat protein, and the tetratricopeptide repeat protein regulates tomato cold resistance [66]. 60S ribosomal protein L6-like (*RPL6*) belongs to the 60S ribosomal subunit family. The protein induced by cold stress, Rbm3, binds to the 60S ribosomal subunit, changes microRNA levels, and enhances overall protein synthesis [67]. In addition, studies have shown that CCT is a cold shock protein in Saccharomyces cerevisiae [68], and Glutaredoxin (Grx) interacts with GR and AhpC to enhance the low-temperature tolerance of the Antarctic psychrophilic bacterium ANT206 [69]. These results mean that these genes may play an important role in low-temperature stress and cold tolerance.

To better understand why LV-T shrimp are more cold-resistant than LV-C shrimp, we conducted a comparative analysis of transcriptomics and methylation groups between LV-T and LV-C shrimp. The analysis found that differentially methylated regions related to genes or differentially expressed genes were significantly enriched in pathways such as the mTOR signaling pathway (ko04150), the cGMP-PKG signaling pathway (ko04022), fatty acid metabolism (ko01212), the TCA cycle (ko00020), and oxidative phosphorylation (ko00190), which are all important parts of the thermogenesis signaling pathway. Studies have shown that the mTOR signaling pathway is involved in the heat stress response [70], fatty acid metabolism can generate heat to resist cold [71], and the TCA cycle can support marine Roseobacter growth at low temperatures [72]. The liver responds to cold exposure through various pathways, including activation of the HSP70/TLR4 signaling pathway, upregulation of RBM3 expression, and increases in glycolysis and glycogen synthesis [73]. In addition, under cold stress, peroxisomal fatty acid catabolism is induced for oxidative phosphorylation, which is the main source of energy production in all tissues under aerobic conditions [74,75]. Various harmful substances are produced in cells under low-temperature stress, and peroxisomes also have detoxification effects, clearing harmful substances through peroxiredoxins, glutathione peroxidases, and catalases [76]. As the main site of drug metabolism, the liver converts lipophilic compounds into more water-soluble compounds through enzymatic action, detoxifying and promoting the excretion of foreign organisms (foreign drugs or chemicals) [77]. Chronic cold exposure induces autophagy, promotes fatty acid oxidation, mitochondrial conversion, and brown fat thermogenesis [78].

**Figure 6:**
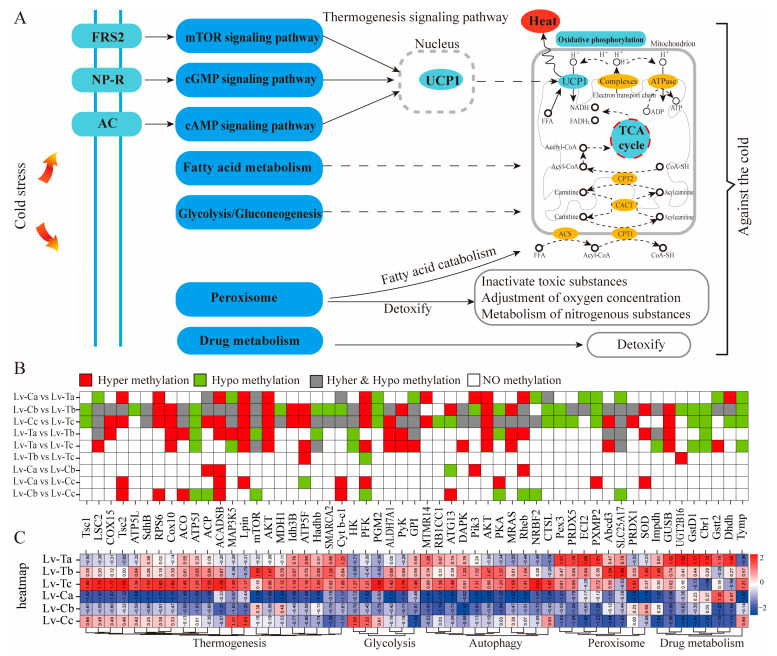
A simple flowchart analyzing the cold resistance of LV-T and LV-C shrimp. (**A**) Thermogenesis-reference pathway and the joint action of related signaling pathways to produce heat; (**B**) represents differentially methylated genes between the two groups, with red indicating hypermethylation, green indicating hypomethylation, gray indicating both hyper and hypomethylation, and white indicating no methylation; and (**C**) corresponds to the differential gene expression shown in B, with values shown as Log2 ratio.

Under cold stress, some genes in pathways such as thermogenesis (*Tsc1*, *LSC2*, mTOR, *MAP3K5*, *Lpin*, *ATP5*, *AKT*, etc.), glycolysis process (*HK*, *PFK*, *PGM2*, *PyK*, *GPI*, etc.), autophagy pathway (*pik3*, *AKT*, *PKA*, *CTSL*, etc.), peroxisome (*Pex3*, *Abcd3*, *SOD*, *PXMP2*, etc.), and drug metabolism (*GUSB*, *GstD1*, *Cbr1*, *Tymp*, etc.) undergo hypermethylation or demethylation, while gene expression differs (Figure 6). We suggest that the DNA methylation in the Pacific white shrimp changes the expression of these genes to adapt to environmental changes and participate in the process of cold resistance, providing new ideas for epigenetics research in cold tolerance. We propose a possible mechanism of cold tolerance in which Pacific white shrimp adjust their overall metabolism, methylation levels, and gene expression to cope with changes in environmental temperature.

## 4. Materials and Methods

### 4.1. L. vannamei Materials and Treatment

*L. vannamei* strains, cold-resistant (Lv-R) and normal (Lv-N), were provided by the Guangxi South American White Shrimp Genetics Breeding Center (China, Guangxi, Fangchenggang Qisha Aquaculture Base). The cold-resistant strain had a significantly higher survival time and rate at 10 °C than the normal strain [61].

To eliminate errors caused by different initial temperatures, both strains were fed under uniform conditions of 28 ± 0.5 °C, salinity of 30%, and pH of 7.9 ± 0.1 for one month before the experiment, with an average weight of 32 g. Shrimps from two families were divided into three groups corresponding to temperature gradients of 28 °C, 18 °C, and 10 °C, cooled down using a cooling system at a rate of 1 °C per hour starting from 28 °C, and samples were taken (40 shrimps from each group) after maintaining the temperature at 18 °C and 10 °C for 24 h. For each condition, there were three biological replicates. In each biological replicate, the hepatopancreases of six shrimps were harvested, mixed, and stored in liquid nitrogen for later use.

### 4.2. Library Construction and Sequencing

Genomic DNA was extracted from the samples (50 mg of hepatopancreas was extracted from each sample). A total of 18 samples were extracted, including 9 from hardy families and 9 from common families, and their concentration and integrity were detected using the NanoPhotometer^®^ spectrophotometer (Implen, Westlake Village, CA, USA) and agarose gel electrophoresis, respectively. Subsequently, sulfuric acid hydrogen sequencing DNA libraries were prepared. In brief, genomic DNA was fragmented into 100–300 bp segments with ultrasound (Covaris, Woburn, MA, USA) and purified using the MiniElute PCR Purification Kit (Qiagen, Germantown, MD, USA). The fragmented DNA was end repaired, and a single “a” nucleotide was added to the 3′ end. The genomic fragments were then connected to methylated sequencing adapters. The adapter-ligated fragments were converted into bisulfite using the ZYMO Methylamp DNA Modification Kit (ZYMO, Irvine, CA, USA), which converts unmethylated cytosines into uracils during sodium bisulfite treatment. DNA end repair and amplification were performed using the NEB library building kit (NEBNext ^®^ Μ Ltra ™ DNA Library Prep Kit for Illumina ^®^, NEB # 7370) with the primers provided in the kit. Finally, the converted DNA fragments were PCR amplified and sequenced using Illumina HiSeqTM 2500 by Gene Denovo Biotechnology Co. (Guangzhou, China).

### 4.3. Data Filtering and Methylation Level Analysis

Data filtering and methylation level analysis raw reads with 10% or more unknown nucleotides (N) and low-quality reads with 40% or more of bases having a quality score ≤20 were filtered out to obtain high-quality clean reads. The clean reads were aligned to the reference genome using BSMAP software (version 2.90) [79]. According to its sequence characteristics, Cytosine (C) can be divided into three forms (CG, CHG, and CHH, where H represents the A, T, or C base). Methylation levels were calculated based on the percentage of methylated cytosines in the genome, chromosome, and different regions (CG, CHG, and CHH) using custom Perl scripts and the correction algorithm described in Lister [80]. To assess different methylation patterns in different genomic regions, the methylation profile at flanking 2 kb regions and gene bodies (or transposable elements) was plotted based on the average methylation levels for each window. The sequencing results were aligned to the *L. vannamei* genome, and the matched sequences were used for subsequent analyses. Subsequent methylation data analyses were performed by taking the average of three biological replicates.

### 4.4. Differentially Methylated Cytosines (DMCs) Analysis

Differentially methylated cytosines (DMCs) analysis: to identify differentially methylated cytosines (DMCs) between two samples, Pearson’s chi-square test (χ2) in methylKit (version: 1.7.10) was used [35]. To identify differentially methylated cytosines (DMCs), the minimum read coverage to call a methylation status for a base was set to 4. Differentially methylated cytosines for each sequence context (CG, CHG, and CHH) between two samples were identified according to different criteria: (1) for CG, the absolute value of the difference in methylation ratio| ≥ 0.25 and q ≤ 0.05; (2) for CHG, the absolute value of the difference in methylation ratio| ≥ 0.25 and q ≤ 0.05; (3) for CHH, the absolute value of the difference in methylation ratio| ≥ 0.15 and q ≤ 0.05; and (4) for all C, the absolute value of the difference in methylation ratio| ≥ 0.2 and q ≤ 0.05. Comparisons between strains at the same temperature were based on the same normal strain as the control group. At different temperatures, comparisons within strains were based on the sample at the highest temperature as the control group.

### 4.5. Differentially Methylated Regions (DMRs) Analysis

To identify differentially methylated regions (DMRs) between two samples, the minimum read coverage to call a methylation status for a base was set to 4. DMRs for each sequence context (CG, CHG, and CHH) according to different criteria: (1) for CG, numbers of GC in each window ≥ 5, absolute value of the difference in methylation ratio ≥ 0.25, and q ≤ 0.05; (2) for CHG, numbers in a window ≥ 5, absolute value of the difference in methylation ratio ≥ 0.25, and q ≤ 0.05; (3) for CHH, numbers in a window≥ 15, absolute value of the difference in methylation ratio ≥ 0.15, and q ≤ 0.05; and (4) for all C, numbers in a window ≥ 20, absolute value of the difference in methylation ratio ≥ 0.2, and q ≤ 0.05. Comparisons between strains at the same temperature were based on the same normal strain as the control group. At different temperatures, comparisons within strains were based on the sample at the highest temperature as the control group.

### 4.6. Functional Enrichment Analysis of DMC/DMR-Related Genes

To analyze the functional enrichment of genes affected by DMCs/DMRs, Gene ontology (GO) enrichment analysis and KEGG pathway enrichment analysis were conducted for DMC/DMR-related genes. GO enrichment analysis provides all GO terms that significantly enriched in genes compared to the genome background and filters out the genes that correspond to biological functions. Firstly, all ceRNAs were mapped to GO terms in the Gene Ontology database (http://www.geneontology.org/, accessed on 3 November 2020), gene numbers were calculated for every term, and significantly enriched GO terms in genes compared to the genome background were defined by a hypergeometric test. Genes usually interact with each other to play roles in certain biological functions. Pathway-based analysis helps to further understand genes biological functions. KEGG is the major public pathway-related database (http://www.kegg.jp/kegg/, accessed on 3 November 2020). Pathway enrichment analysis identified significantly enriched metabolic pathways or signal transduction pathways in genes compared with the whole genome background.

### 4.7. RNA Extraction, Library Construction, Sequencing, and Transcriptome Profiling Analysis

Total RNA was extracted using the Trizol reagent kit (Invitrogen, Carlsbad, CA, USA) according to the manufacturer’s protocol. RNA quality was assessed on an Agilent 2100 Bioanalyzer (Agilent Technologies, Palo Alto, CA, USA) and checked using RNase-free agarose gel electrophoresis. A total of 100 mg of hepatopancreas was extracted from each sample. A total of 18 samples were extracted, including 9 from hardy families and 9 from common families. After total RNA was extracted, eukaryotic mRNA was enriched by Oligo(dT) beads. Then the enriched mRNA was fragmented into short fragments using fragmentation buffer and reverse transcribed into cDNA with random primers. Second-strand cDNA was synthesized with DNA polymerase I, RNase H, dNTP, and buffer using the NEBNext Ultra RNA Library Prep Kit (NEB#7530, New England Biolabs, Ipswich, MA, USA). Then the cDNA fragments were purified with the QiaQuick PCR extraction kit (Qiagen, Venlo, The Netherlands), end repaired, poly(A) added, and ligated to Illumina sequencing adapters. The ligation products were size-selected by agarose gel electrophoresis, PCR amplified, and sequenced using Illumina HiSeq2500 by Gene Denovo Biotechnology Co. (Guangzhou, China).

Reads obtained from the sequencing machines include raw reads containing adapters or low-quality bases, which will affect the following assembly and analysis. Thus, to get high-quality, clean reads, reads were further filtered by fastp [36] (version 0.18.0). The short read alignment tool Bowtie2 [37] (version) was used for mapping reads to the ribosome RNA (rRNA) database. The rRNA-mapped reads will then be removed. The remaining clean reads were further used in assembly and gene abundance calculations. An index of the reference genome was built, and paired-end clean reads were mapped to the reference genome using HISAT2. 2.4 [81] with “rna strandness RF” and other parameters set as default. The mapped reads of each sample were assembled by using StringTie v1.3.1 [82,83] in a reference-based approach. For each transcription region, a FPKM (fragment per kilobase of transcript per million mapped reads) value was calculated to quantify its expression abundance and variations using StringTie v1.3.1 software. RNA differential expression analysis was performed by DESeq2 v1.20.0 [84] software between two different groups (and by edgeR [85] between two samples). The genes/transcripts with the parameter of false discovery rate (FDR) below 0.05 and an absolute fold change ≥2 were considered differentially expressed genes/transcripts.

### 4.8. Correlation of DNA Methylation and Gene Expression in Samples

To determine whether gene expression influences DNA methylation in a sample, genes were classified into four classes based on their expression levels: a non-expressed group (RPKM (reads per kilobase per million reads mapped ≤1), a low-expressed group (1 < RPKM < 10), a middle-expressed group (10 < RPKM < 100), and a high-expressed group (RPKM > 100)). To analyze whether DNA methylation influences gene expression in a sample, genes were classified into four classes according to their methylation level, including a non-methylation group, a low-methylation group, a middle-methylation group, and a high-methylation group (genes excluded from non-methylation were divided into 3 groups on average). Spearman correlation analysis was performed to discern statistically the relationships between DNA methylation and gene expression within the ±2 kb flanking regions and gene body regions. Rho > 0, means positive correlation; Rho < 0, means negative correlation. To analyze whether differentially expressed genes (DEGs) influence DNA methylation between groups, DEGs were categories four classes based on their different expression patterns: a special-down group (genes specifically expressed in the control group), a special-up group (genes specifically expressed in the treatment group), an other-down group (genes downregulated expressed in the treatment group), and an other-up group (genes upregulated expressed in the treatment group). To determine whether DNA methylation level in differentially methylated regions (DMRs) influences gene expression between groups, genes were classified according to genomic location, including the ±2 kb flanking regions and gene body regions.

### 4.9. Correlation of DMRs and DEGs between Groups

To explore the potential functions of DNA methylation responsible for DEGs, common genes between DMR-related genes and DEGs were analyzed, as well as Gene ontology (GO) enrichment analysis and KEGG pathway enrichment analysis for DEGs with DMRs. GO enrichment analysis provides all GO terms that significantly enrich genes compared to the genome background and filters out the genes that correspond to biological functions. Firstly, DEGs with DMRs were mapped to GO terms in the Gene Ontology database (http://www.geneontology.org/, accessed on 3 November 2020), gene numbers were calculated for every term, and significantly enriched GO terms in genes compared to the genome background were defined by a hypergeometric test. Genes usually interact with each other to play roles in certain biological functions. Pathway-based analysis helps to further understand genes biological functions. KEGG is the major public pathway-related database (http://www.kegg.jp/kegg/, accessed on 3 November 2020). Pathway enrichment analysis identified significantly enriched metabolic pathways or signal transduction pathways in genes compared with the whole genome background.

## Figures and Tables

**Table 1:** Summary of genome-wide methylation sequencing data. Mapped Reads: the number of reads that were mapped to the reference genome for each sample. Mapped Ratio (%): the mapping rate of each sample relative to the reference genome. Sequence Depth: the sequencing depth of each sample relative to the reference genome, calculated as reads × reads_length/genome_size. Lv-Ca is the control group of the normal strain at 28 °C (Lv-C28), Lv-Cb is the stress group of the normal strain at 18 °C (Lv-C18), and Lv-Cc is the low-temperature stress group of the normal strain at 10 °C (Lv-C10). Lv-Ta is the control group of the cold-resistant strain at 28 °C (Lv-T28), Lv-Tb is the stress group of the cold-resistant strain at 1 °C (Lv-T18), and Lv-Tc is the low-temperature stress group of the cold-resistant strain at 10 °C (Lv-T10). Each group has three replicates.

Samples	Raw Reads	Clean Reads	Mapped Reads	Mapped Ratio (%)	Sequence Depth	Bisulfite Conversion Efficiency Ratio (%)
Lv-Ca-1	59,995,237,800	378,769,935	274,911,219	72.58	24.79	99.07%
Lv-Ca-2	54,147,382,350	343,619,289	255,996,371	74.5	23.08	98.79%
Lv-Ca-3	50,212,993,650	317,781,300	222,383,354	69.98	20.05	99.28%
Lv-Cb-1	51,239,241,000	333,260,024	240,747,042	72.24	21.71	99.09%
Lv-Cb-2	53,152,604,100	339,290,790	248,360,859	73.2	22.39	99.32%
Lv-Cb-3	62,313,481,650	400,634,145	291,060,707	72.65	26.24	99.09%
Lv-Cc-1	52,176,070,350	334,483,395	240,627,355	71.94	21.7	99.09%
Lv-Cc-2	52,775,231,700	335,017,171	248,649,745	74.22	22.42	98.98%
Lv-Cc-3	52,397,810,100	338,734,377	251,408,655	74.22	22.67	99.22%
Lv-Ta-1	45,925,674,750	287,311,022	200,112,127	69.65	18.04	99.37%
Lv-Ta-2	43,817,521,500	270,324,896	188,632,713	69.78	17.01	99.15%
Lv-Ta-3	51,239,241,000	333,260,024	240,747,042	72.24	21.71	99.06%
Lv-Tb-1	60,521,113,350	389,231,454	290,055,280	74.52	26.15	99.18%
Lv-Tb-2	54,667,904,100	352,826,654	263,949,620	74.81	23.8	99.08%
Lv-Tb-3	64,194,630,150	414,526,126	300,531,442	72.5	27.1	98.86%
Lv-Tc-1	54,767,071,950	353,430,171	263,906,309	74.67	23.8	98.99%
Lv-Tc-2	58,237,244,250	375,319,627	276,873,289	73.77	24.96	98.91%
Lv-Tc-3	53,603,465,250	347,707,812	258,903,237	74.46	23.34	98.73%

**Table 2:** Information on DMR-related genes and DEGs.

Common Groups	Gene ID	Symbol	Hyper/Hypo	Region	Context	Expression	Putative Function
Lv-Ca vs. Lv-Ta	LOC113804428	*Rpn2*	Hyper	Gene body/Downstream	CG	Up	26S proteasome non-ATPase regulatory subunit 1-like.
Lv-Cb vs. Lv-Tb	MSTRG.717	*Hsd17b4*	Hypo	Downstream2kb	CG	Up	Peroxisomal multifunctional enzyme type 2.
Lv-Cc vs. Lv-Tc	LOC113829947	*SdhD*	Hypo	Downstream2kb	CG	Up	Succinate dehydrogenase [ubiquinone] cytochrome b small subunit.
	LOC113809689	*Tomm22*	Hyper/hypo	Gene body	CG/CHG	Up	Mitochondrial import receptor subunit TOM22 homolog.
	LOC113809406	*RAI14*	Hyper/hypo	Gene body/Downstream	CG/CHH	Up	Ankycorbin-like.
Lv-Ca vs. Lv-Ta	LOC113811306	*CACNA1S*	Hypo	Gene body	CG/CHH	Down	Muscle calcium channel subunit alpha-1-like.
Lv-Cb vs. Lv-Tb	LOC113805157	*ATP8B2*	Hyper	Gene body	CG	Up	Phospholipid-transporting ATPase ID-like.
	LOC113817591	*ttc39b*	Hypo	Gene body	CG	Up	Tetratricopeptide repeat protein 39B-like.
Lv-Ca vs. Lv-Ta	LOC113817695	*MagR*	Hyper	Gene body	CG	Up	Balbiani ring protein 3-like.
Lv-Cc vs. Lv-Tc	LOC113815521	*NCX1*	Hypo	Gene body	CG	Up	Sodium/calcium exchanger regulatory protein 1-like.
	LOC113826211	*eif3k*	Hyper	Upstream2kb/Gene body	CG	Up	Eukaryotic translation initiation factor 3 subunit 12.
	LOC113824113	*brm*	Hyper/hypo	Gene body/Downstream	CG	Up	ATP-dependent helicase brm-like.
Lv-Cb vs. Lv-Tb	LOC113822938	*eIF2alpha*	Hyper	Gene body/Downstream	CG	Down	Eukaryotic translation initiation factor 2 subunit 1-like.
Lv-Cc vs. Lv-Tc	LOC113826494	*RPL6*	Hyper	Downstream	CG	Down	60S ribosomal protein L6-like.
	LOC113829948	*SdhD*	Hyper/hypo	Gene body	CG	Down	Succinate dehydrogenase [ubiquinone] cytochrome b small subunit.
	LOC113805669	*cct3*	Hypo	Gene body	CG	Down	T-complex protein 1 subunit gamma-like.
	LOC113816677	*smim8*	Hypo	Gene body	CG	Down	Small integral membrane protein 8-like.
	LOC113828991	*AK3*	Hypo	Gene body	CG	Down	GTP:AMP phosphotransferase AK3.
	LOC113824689	*DDX46*	Hypo	Gene body	CG	Down	Probable ATP-dependent RNA helicase DDX46.
	MSTRG.9247	*iolG*	Hypo	Gene body	CG	Down	Uncharacterized oxidoreductase YjhC-like.
	LOC113823684	*Riok2*	Hypo	Gene body	CG	Down	Serine/threonine-protein kinase RIO2-like.
	LOC113829948	*SdhD*	Hypo	Gene body	CG	Down	Succinate dehydrogenase [ubiquinone] cytochrome b small subunit.
	LOC113806157	*ISCU*	Hyper	Gene body	CG	Up	Iron-sulfur cluster assembly 1 homolog.
	LOC113807917	*SAT2*	Hyper	Gene body/Downstream	CG	Up	Spermidine/spermine N(1)-acetyltransferase-like protein 1.
	MSTRG.1468	*mon2*	Hyper	Upstream2kb	CG	Up	Protein MON2 homolog.
	LOC113828355	*GUSB*	Hyper	Gene body	CG/CHH	Up	Beta-glucuronidase-like.
	LOC113828992	*AK3*	Hypo	Gene body	CG	Up	GTP:AMP phosphotransferase AK3.
	MSTRG.15122	*LMAN1*	Hypo	Gene body/Downstream	CG	Up	ERGIC-53.
	LOC113803175	*VPS11*	Hypo	Gene body/Downstream	CG	Up	Vacuolar protein sorting-associated protein 11 homolog.
	LOC113818707	*dhtkd1*	Hyper	Gene body	CG	Up	Probable 2-oxoglutarate dehydrogenase E1 component DHKTD1.
	LOC113825518	*NDUFS3*	Hyper	Gene body/Downstream	CG	Up	NADH dehydrogenase [ubiquinone] iron-sulfur protein 3.
	LOC113829947	*SdhD*	Hyper	Gene body	CG	Up	Succinate dehydrogenase [ubiquinone] cytochrome b small subunit.
	LOC113804844	*Sin3a*	Hyper/hypo	Gene body/Downstream	CG	Up	Paired amphipathic helix protein Sin3a.
	LOC113814290	*Grx3*	Hyper/hypo	Gene body/Downstream	CG	Up	Glutaredoxin-3.
	LOC113823427		Hyper/hypo	Gene body	CG/CHG/CHH	Up	Uncharacterized protein.

## Data Availability

The methylome and transcriptome data were deposited in the NCBI BioProject under accession numbers PRJNA974495 and PRJNA974093, respectively.

## Supplementary Materials

The following supporting information can be downloaded at: https://www.mdpi.com/article/10.3390/ijms241411573/s1.

## References

[1] Baerwald M.R., Meek M.H., Stephens M.R., Nagarajan R.P., Goodbla A.M., Tomalty K.M.H., Thorgaard G.H., May B., Nichols K.M. (2016). Migration-related phenotypic divergence is associated with epigenetic modifications in rainbow trout. Mol. Ecol..

[2] Wilschut R.A., Oplaat C., Snoek L.B., Kirschner J., Verhoeven K.J. (2016). Natural epigenetic variation contributes to heritable flowering divergence in a widespread asexual dandelion lineage. Mol. Ecol..

[3] Zhang Y.Y., Fischer M., Colot V., Bossdorf O. (2013). Epigenetic variation creates potential for evolution of plant phenotypic plasticity. New Phytol..

[4] Johannes F., Porcher E., Teixeira F.K., Saliba-Colombani V., Simon M., Agier N., Bulski A., Albuisson J., Heredia F., Audigier P. (2009). Assessing the impact of transgenerational epigenetic variation on complex traits. PLoS Genet..

[5] Mirouze M., Paszkowski J. (2011). Epigenetic contribution to stress adaptation in plants. Curr. Opin. Plant Biol..

[6] Cox B.D. (2013). On the difficulty in getting out of historical ruts: Waddington and an argument for behavioral epigenetics. New Ideas Psychol..

[7] Bonasio R., Li Q., Lian J., Mutti N.S., Jin L., Zhao H., Zhang P., Wen P., Xiang H., Ding Y. (2012). Genome-wide and caste-specific DNA methylomes of the ants Camponotus floridanus and Harpegnathos saltator. Curr. Biol..

[8] Schübeler D. (2015). Function and information content of DNA methylation. Nature.

[9] Aniagu S.O., Williams T.D., Allen Y., Katsiadaki I., Chipman J.K. (2008). Global genomic methylation levels in the liver and gonads of the three-spine stickleback (*Gasterosteus aculeatus*) after exposure to hexabromocyclododecane and 17-beta oestradiol. Environ. Int..

[10] Goll M.G., Bestor T.H. (2005). Eukaryotic cytosine methyltransferases. Annu. Rev. Biochem..

[11] Bird A. (2002). DNA methylation patterns and epigenetic memory. Genes. Dev..

[12] Cedar H., Bergman Y. (2012). Programming of DNA methylation patterns. Annu. Rev. Biochem..

[13] Wang Y., Wang T., Qiao L., Zhu J., Fan J., Zhang T., Wang Z.X., Li W., Chen A., Huang B. (2017). DNA methyltransferases contribute to the fungal development, stress tolerance and virulence of the entomopathogenic fungus Metarhizium robertsii. Appl. Microbiol. Biotechnol..

[14] Jaenisch R., Bird A. (2003). Epigenetic regulation of gene expression: How the genome integrates intrinsic and environmental signals. Nat. Genet..

[15] Secco D., Wang C., Shou H., Schultz M.D., Chiarenza S., Nussaume L., Ecker J.R., Whelan J., Lister R. (2015). Stress induced gene expression drives transient DNA methylation changes at adjacent repetitive elements. eLife.

[16] Boyko A., Kovalchuk I. (2008). Epigenetic control of plant stress response. Environ. Mol. Mutagen..

[17] Hawes N.A., Tremblay L.A., Pochon X., Dunphy B., Fidler A.E., Smith K.F. (2018). Effects of temperature and salinity stress on DNA methylation in a highly invasive marine invertebrate, the colonial ascidian Didemnum vexillum. PeerJ.

[18] Metzger D.C.H., Schulte P.M. (2017). Persistent and plastic effects of temperature on DNA methylation across the genome of threespine stickleback (*Gasterosteus aculeatus*). Proc. R. Soc. B Biol. Sci..

[19] Anastasiadi D., Díaz N. (2017). Small ocean temperature increases elicit stage-dependent changes in DNA methylation and gene expression in a fish, the European sea bass. Sci. Rep..

[20] Han B., Li W., Chen Z., Xu Q., Luo J., Shi Y., Li X., Yan X., Zhang J. (2016). Variation of DNA Methylome of Zebrafish Cells under Cold Pressure. PLoS ONE.

[21] Li S., He F., Wen H., Si Y., Liu M., Huang Y., Wu S. (2020). Half Smooth Tongue Sole (*Cynoglossus semilaevis*) Under Low Salinity Stress Can Change Hepatic igf2 Expression Through DNA Methylation. J. Ocean. Univ. China.

[22] Navarro-Martín L., Viñas J., Ribas L., Díaz N., Gutiérrez A., Di Croce L., Piferrer F. (2011). DNA methylation of the gonadal aromatase (cyp19a) promoter is involved in temperature-dependent sex ratio shifts in the European sea bass. PLoS Genet..

[23] Venegas D., Marmolejo-Valencia A., Valdes-Quezada C., Govenzensky T., Recillas-Targa F., Merchant-Larios H. (2016). Dimorphic DNA methylation during temperature-dependent sex determination in the sea turtle Lepidochelys olivacea. Gen. Comp. Endocrinol..

[24] Varriale A., Bernardi G. (2006). DNA methylation and body temperature in fishes. Gene.

[25] Jabbari K., Cacciò S., Païs de Barros J.P., Desgrès J., Bernardi G. (1997). Evolutionary changes in CpG and methylation levels in the genome of vertebrates. Gene.

[26] de la Peña M.V., Piskobulu V., Murgatroyd C., Hager R. (2021). DNA methylation patterns respond to thermal stress in the viviparous cockroach Diploptera punctata. Epigenetics.

[27] Williamson S.M., Ingelson-Filpula W.A., Hadj-Moussa H., Storey K.B. (2021). Epigenetic underpinnings of freeze avoidance in the goldenrod gall moth, Epiblema scudderiana. J. Insect Physiol..

[28] Chen P., Xiao W.F., Pan M.H., Xiao J.S., Feng Y.J., Dong Z.Q., Zou B.X., Zhou L., Zhang Y.H., Lu C. (2020). Comparative genome-wide DNA methylation analysis reveals epigenomic differences in response to heat-humidity stress in Bombyx mori. Int. J. Biol. Macromol..

[29] Li B., Hou L., Zhu D., Xu X., An S., Wang X. (2018). Identification and caste-dependent expression patterns of DNA methylation associated genes in Bombus terrestris. Sci. Rep..

[30] Sun D., Li Q., Yu H. (2022). DNA methylation differences between male and female gonads of the oyster reveal the role of epigenetics in sex determination. Gene.

[31] Trigg S.A., Venkataraman Y.R., Gavery M.R., Roberts S.B., Bhattacharya D., Downey-Wall A., Eirin-Lopez J.M., Johnson K.M., Lotterhos K.E., Puritz J.B. (2022). Invertebrate methylomes provide insight into mechanisms of environmental tolerance and reveal methodological biases. Mol. Ecol. Resour..

[32] Hearn J., Pearson M., Blaxter M., Wilson P.J., Little T.J. (2019). Genome-wide methylation is modified by caloric restriction in Daphnia magna. BMC Genom..

[33] Zeng S., Huang Z., Hou D., Liu J., Weng S., He J. (2017). Composition, diversity and function of intestinal microbiota in pacific white shrimp (*Litopenaeus vannamei*) at different culture stages. PeerJ.

[34] Ponce-Palafox J., Martinez-Palacios C.A., Ross L.G. (1997). The effects of salinity and temperature on the growth and survival rates of juvenile white shrimp, *Penaeus vannamei*, Boone, 1931. Aquaculture.

[35] Akalin A., Kormaksson M., Li S., Garrett-Bakelman F.E., Figueroa M.E., Melnick A., Mason C.E. (2012). methylKit: A comprehensive R package for the analysis of genome-wide DNA methylation profiles. Genome Biol..

[36] Chen S., Zhou Y., Chen Y., Gu J. (2018). fastp: An ultra-fast all-in-one FASTQ preprocessor. Bioinformatics.

[37] Langmead B., Salzberg S.L. (2012). Fast gapped-read alignment with Bowtie 2. Nat. Methods.

[38] Razin A., Kantor B. (2005). DNA methylation in epigenetic control of gene expression. Prog. Mol. Subcell. Biol..

[39] Sahu P.P., Pandey G., Sharma N., Puranik S., Muthamilarasan M., Prasad M. (2013). Epigenetic mechanisms of plant stress responses and adaptation. Plant. Cell Rep..

[40] Liu T., Li Y., Duan W., Huang F., Hou X. (2017). Cold acclimation alters DNA methylation patterns and confers tolerance to heat and increases growth rate in Brassica rapa. J. Exp. Bot..

[41] Hao Y., Cui Y., Gu X. (2016). Genome-wide DNA methylation profiles changes associated with constant heat stress in pigs as measured by bisulfite sequencing. Sci. Rep..

[42] Huang H., Wu P., Zhang S., Shang Q., Yin H., Hou Q., Zhong J., Guo X. (2019). DNA methylomes and transcriptomes analysis reveal implication of host DNA methylation machinery in BmNPV proliferation in Bombyx mori. BMC Genom..

[43] Krauss V., Eisenhardt C., Unger T. (2009). The genome of the stick insect Medauroidea extradentata is strongly methylated within genes and repetitive DNA. PLoS ONE.

[44] Zemach A., McDaniel I.E., Silva P., Zilberman D. (2010). Genome-wide evolutionary analysis of eukaryotic DNA methylation. Science.

[45] Yang H., Chang F., You C., Cui J., Zhu G., Wang L., Zheng Y., Qi J., Ma H. (2015). Whole-genome DNA methylation patterns and complex associations with gene structure and expression during flower development in Arabidopsis. Plant J. For. Cell Mol. Biol..

[46] Brenet F., Moh M., Funk P., Feierstein E., Viale A.J., Socci N.D., Scandura J.M. (2011). DNA methylation of the first exon is tightly linked to transcriptional silencing. PLoS ONE.

[47] Wang H., Beyene G., Zhai J., Feng S., Fahlgren N., Taylor N.J., Bart R., Carrington J.C., Jacobsen S.E., Ausin I. (2015). CG gene body DNA methylation changes and evolution of duplicated genes in cassava. Proc. Natl. Acad. Sci. USA.

[48] Yang Y., Liang G., Niu G., Zhang Y., Zhou R., Wang Y., Mu Y., Tang Z., Li K. (2017). Comparative analysis of DNA methylome and transcriptome of skeletal muscle in lean-, obese-, and mini-type pigs. Sci. Rep..

[49] Song M., He Y., Zhou H., Zhang Y., Li X., Yu Y. (2016). Combined analysis of DNA methylome and transcriptome reveal novel candidate genes with susceptibility to bovine Staphylococcus aureus subclinical mastitis. Sci. Rep..

[50] Chan S.W., Henderson I.R., Jacobsen S.E. (2005). Gardening the genome: DNA methylation in Arabidopsis thaliana. Nat. Rev. Genet..

[51] Li X., Zhu J., Hu F., Ge S., Ye M., Xiang H., Zhang G., Zheng X., Zhang H., Zhang S. (2012). Single-base resolution maps of cultivated and wild rice methylomes and regulatory roles of DNA methylation in plant gene expression. BMC Genom..

[52] Xu J., Zhou S., Gong X., Song Y., van Nocker S., Ma F., Guan Q. (2018). Single-base methylome analysis reveals dynamic epigenomic differences associated with water deficit in apple. Plant Biotechnol. J..

[53] Marshall H., Lonsdale Z.N., Mallon E.B. (2019). Methylation and gene expression differences between reproductive and sterile bumblebee workers. Evol. Lett..

[54] Patalano S., Vlasova A., Wyatt C., Ewels P., Camara F., Ferreira P.G., Asher C.L., Jurkowski T.P., Segonds-Pichon A., Bachman M. (2015). Molecular signatures of plastic phenotypes in two eusocial insect species with simple societies. Proc. Natl. Acad. Sci. USA.

[55] Glastad K.M., Gokhale K., Liebig J., Goodisman M.A. (2016). The caste- and sex-specific DNA methylome of the termite Zootermopsis nevadensis. Sci. Rep..

[56] Libbrecht R., Oxley P.R., Keller L., Kronauer D.J. (2016). Robust DNA Methylation in the Clonal Raider Ant Brain. Curr. Biol..

[57] Foret S., Kucharski R., Pittelkow Y., Lockett G.A., Maleszka R. (2009). Epigenetic regulation of the honey bee transcriptome: Unravelling the nature of methylated genes. BMC Genom..

[58] Lyko F., Foret S., Kucharski R., Wolf S., Falckenhayn C., Maleszka R. (2010). The honey bee epigenomes: Differential methylation of brain DNA in queens and workers. PLoS Biol..

[59] Provataris P., Meusemann K., Niehuis O., Grath S., Misof B. (2018). Signatures of DNA Methylation across Insects Suggest Reduced DNA Methylation Levels in Holometabola. Genome Biol. Evol..

[60] Zilberman D. (2017). An evolutionary case for functional gene body methylation in plants and animals. Genome Biol..

[61] Zhu W., Yang C., Chen X., Liu Q., Li Q., Peng M., Wang H., Chen X., Yang Q., Liao Z. (2021). Single-Cell Ribonucleic Acid Sequencing Clarifies Cold Tolerance Mechanisms in the Pacific White Shrimp (*Litopenaeus vannamei*). Front. Genet..

[62] Papaevgeniou N., Chondrogianni N. (2014). The ubiquitin proteasome system in Caenorhabditis elegans and its regulation. Redox Biol..

[63] Peng Y.F., Mandai K., Sakisaka T., Okabe N., Yamamoto Y., Yokoyama S., Mizoguchi A., Shiozaki H., Monden M., Takai Y. (2000). Ankycorbin: A novel actin cytoskeleton-associated protein. Genes Cells.

[64] Bertolin G., Ferrando-Miguel R., Jacoupy M., Traver S., Grenier K., Greene A.W., Dauphin A., Waharte F., Bayot A., Salamero J. (2013). The TOMM machinery is a molecular switch in PINK1 and PARK2/PARKIN-dependent mitochondrial clearance. Autophagy.

[65] Honsho M., Mawatari S., Fujiki Y. (2022). ATP8B2-Mediated Asymmetric Distribution of Plasmalogens Regulates Plasmalogen Homeostasis and Plays a Role in Intracellular Signaling. Front. Mol. Biosci..

[66] Zhang Y., Peng Y. (2023). Tetratricopeptide repeat protein SlREC2 positively regulates cold tolerance in tomato. Plant Physiol..

[67] Dresios J., Aschrafi A., Owens G.C., Vanderklish P.W., Edelman G.M., Mauro V.P. (2005). Cold stress-induced protein Rbm3 binds 60S ribosomal subunits, alters microRNA levels, and enhances global protein synthesis. Proc. Natl. Acad. Sci. USA.

[68] Somer L., Shmulman O., Dror T., Hashmueli S., Kashi Y. (2002). The eukaryote chaperonin CCT is a cold shock protein in Saccharomyces cerevisiae. Cell Stress Chaperones.

[69] Wang Y., Wang Q., Hou Y., Liu J. (2022). Glutaredoxin Interacts with GR and AhpC to Enhance Low-Temperature Tolerance of Antarctic Psychrophile Psychrobacter sp. ANT206. Int. J. Mol. Sci..

[70] Xu J., Strasburg G.M. (2022). Thermal stress affects proliferation and differentiation of turkey satellite cells through the mTOR/S6K pathway in a growth-dependent manner. PLoS ONE.

[71] Blondin D.P., Tingelstad H.C., Noll C., Frisch F., Phoenix S., Guérin B., Turcotte É.E., Richard D., Haman F., Carpentier A.C. (2017). Dietary fatty acid metabolism of brown adipose tissue in cold-acclimated men. Nat. Commun..

[72] Wang M., Wang H., Wang P., Fu H.H., Li C.Y. (2022). TCA cycle enhancement and uptake of monomeric substrates support growth of marine Roseobacter at low temperature. Nat. Commun..

[73] Shi H., Yao R., Lian S., Liu P., Liu Y., Yang Y.Y., Yang H., Li S. (2019). Regulating glycolysis, the TLR4 signal pathway and expression of RBM3 in mouse liver in response to acute cold exposure. Stress.

[74] Forner F., Foster L.J., Campanaro S., Valle G., Mann M. (2006). Quantitative proteomic comparison of rat mitochondria from muscle, heart, and liver. Mol. Cell Proteom..

[75] D’Elia D., Catalano D., Licciulli F., Turi A., Tripoli G., Porcelli D., Saccone C., Caggese C. (2006). The MitoDrome database annotates and compares the OXPHOS nuclear genes of Drosophila melanogaster, Drosophila pseudoobscura and Anopheles gambiae. Mitochondrion.

[76] Zhu X., Chen Z., Shen W., Huang G., Sedivy J.M., Wang H., Ju Z. (2021). Inflammation, epigenetics, and metabolism converge to cell senescence and ageing: The regulation and intervention. Signal Transduct. Target. Ther..

[77] McDonnell A.M., Dang C.H. (2013). Basic review of the cytochrome p450 system. J. Adv. Pract. Oncol..

[78] Yau W.W., Wong K.A., Zhou J., Thimmukonda N.K., Wu Y., Bay B.H., Singh B.K., Yen P.M. (2021). Chronic cold exposure induces autophagy to promote fatty acid oxidation, mitochondrial turnover, and thermogenesis in brown adipose tissue. iScience.

[79] Xi Y., Li W. (2009). BSMAP: Whole genome bisulfite sequence MAPping program. BMC Bioinform..

[80] Lister R., Pelizzola M., Dowen R.H., Hawkins R.D., Hon G., Tonti-Filippini J., Nery J.R., Lee L., Ye Z., Ngo Q.M. (2009). Human DNA methylomes at base resolution show widespread epigenomic differences. Nature.

[81] Kim D., Langmead B., Salzberg S.L. (2015). HISAT: A fast spliced aligner with low memory requirements. Nat. Methods.

[82] Pertea M., Kim D., Pertea G.M., Leek J.T., Salzberg S.L. (2016). Transcript-level expression analysis of RNA-seq experiments with HISAT, StringTie and Ballgown. Nat. Protoc..

[83] Pertea M., Pertea G.M., Antonescu C.M., Chang T.C., Mendell J.T., Salzberg S.L. (2015). StringTie enables improved reconstruction of a transcriptome from RNA-seq reads. Nat. Biotechnol..

[84] Love M.I., Huber W., Anders S. (2014). Moderated estimation of fold change and dispersion for RNA-seq data with DESeq2. Genome Biol..

[85] Robinson M.D., McCarthy D.J., Smyth G.K. (2010). edgeR: A Bioconductor package for differential expression analysis of digital gene expression data. Bioinformatics.

