# Supplementary material for: Integrated Analysis of DNA Methylome and Transcriptome Reveals Epigenetic Regulation of Cold Tolerance in Litopenaeus vannamei
Source: Int J Mol Sci. 2023 Jul 18;24(14):11573. doi: 10.3390/ijms241411573 (PMC10380378; doi:10.3390/ijms241411573)
Supplement: Supplementary file 1 [file ijms-24-11573-s001.zip › ijms-2456862-supplementary figures.pdf]

Supplementary figures

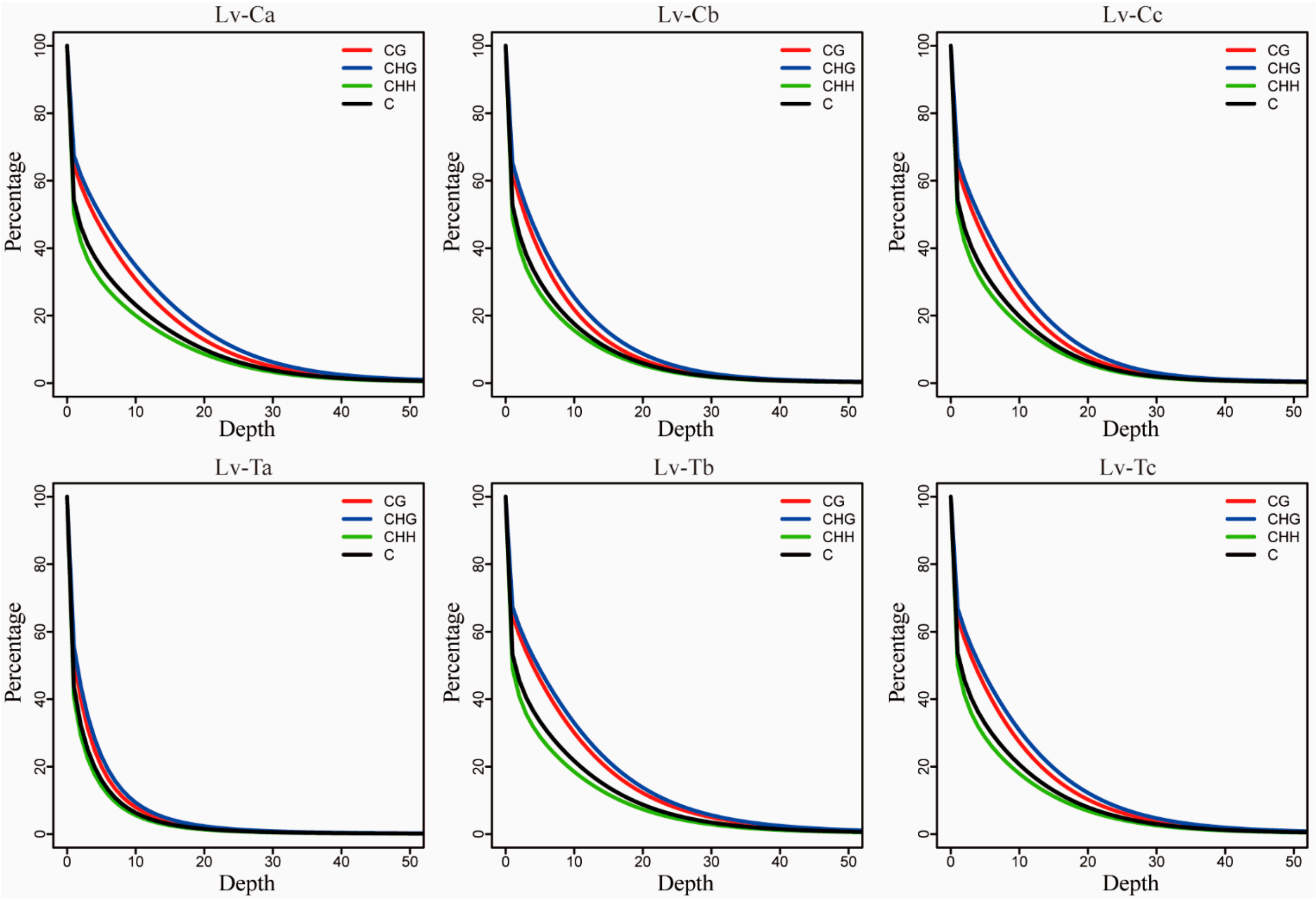

Supplementary Figure S1. The sequencing depth and saturation in Lv-T and Lv-C in response to cold stress. Lv-T represents the cold-tolerant strain, and Lv-C represents the common strain. Lv-Ta and Lv-Ca are control groups at a constant temperature of 28℃, Lv-Tb and Lv-Cb are treatment groups at 18℃, and Lv-Tc and Lv-Cc are treatment groups at a low-temperature stress of 10℃.

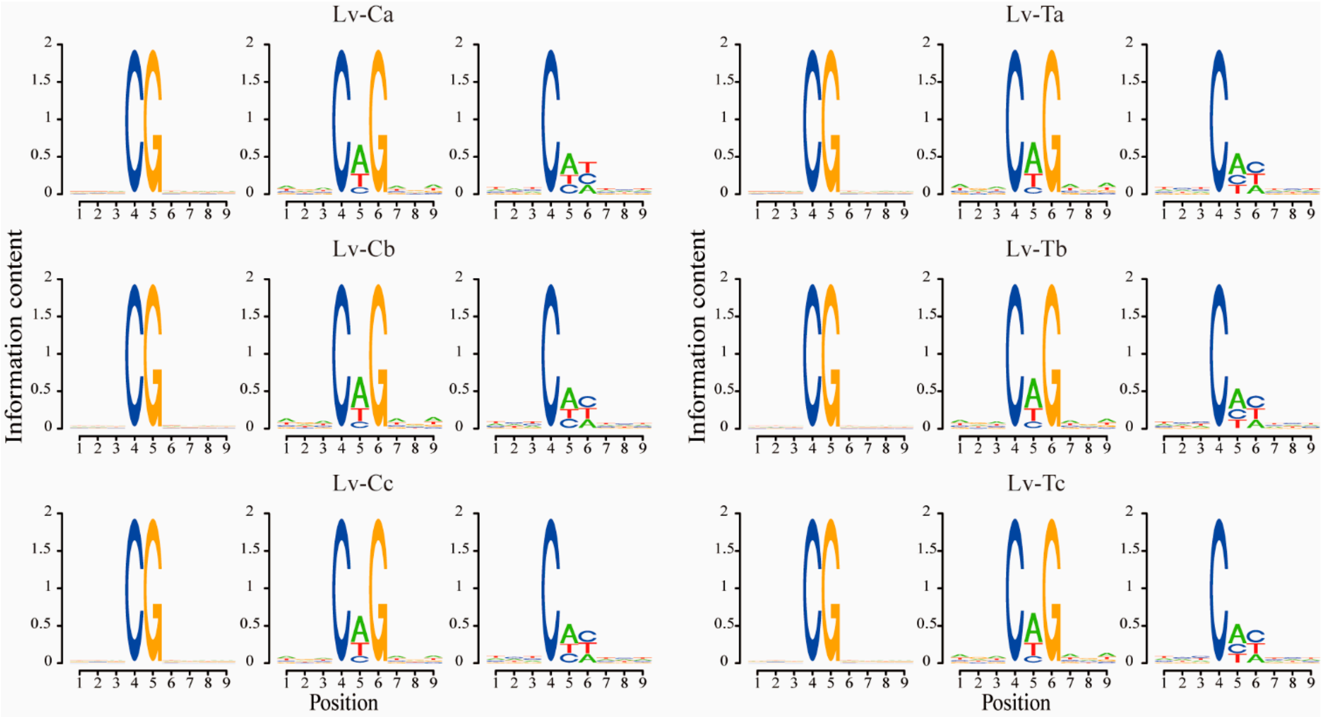

Supplementary Figure S2. DNA sequence logo plot of the methylated cytosine contexts. Information was collected for the 9 base pair bases around the position of the methylated cytosine contexts. The x-axis of the graph represents the base position, where the fourth position is used to analyze the C base. The y-axis represents the entropy value (0 being the minimum, indicating a 25% distribution for each of the four bases and 2 being the maximum, indicating an uneven distribution for the four bases, with one specific base occurring most frequently).

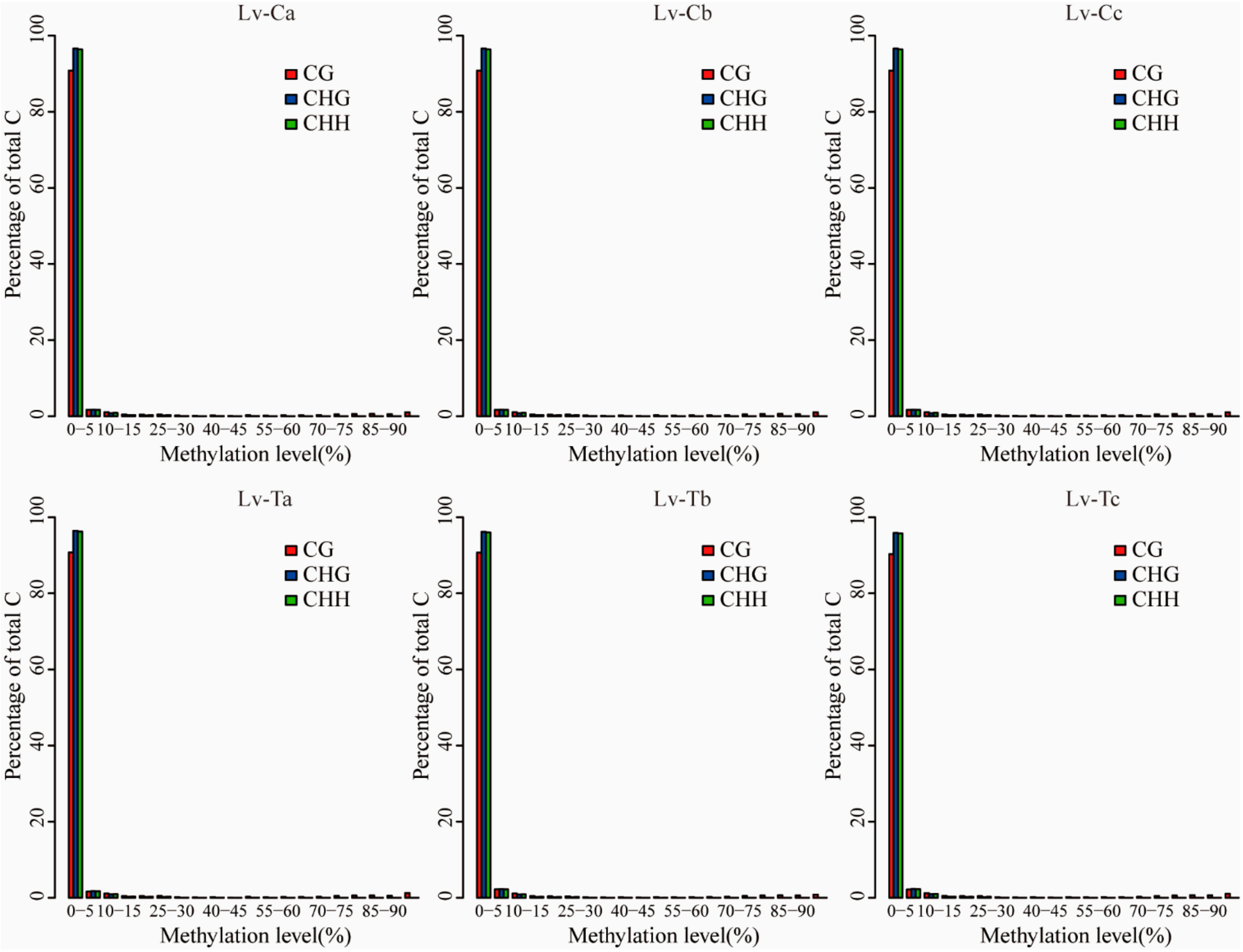

Supplementary Figure S3. Distribution plot of methylation levels. The x-axis of the figure represents the level of methylation, ranging from 0% to 100%, with each category consisting of a 10% range. The y-axis represents the proportion of Cs at a specific level of methylation among all Cs.

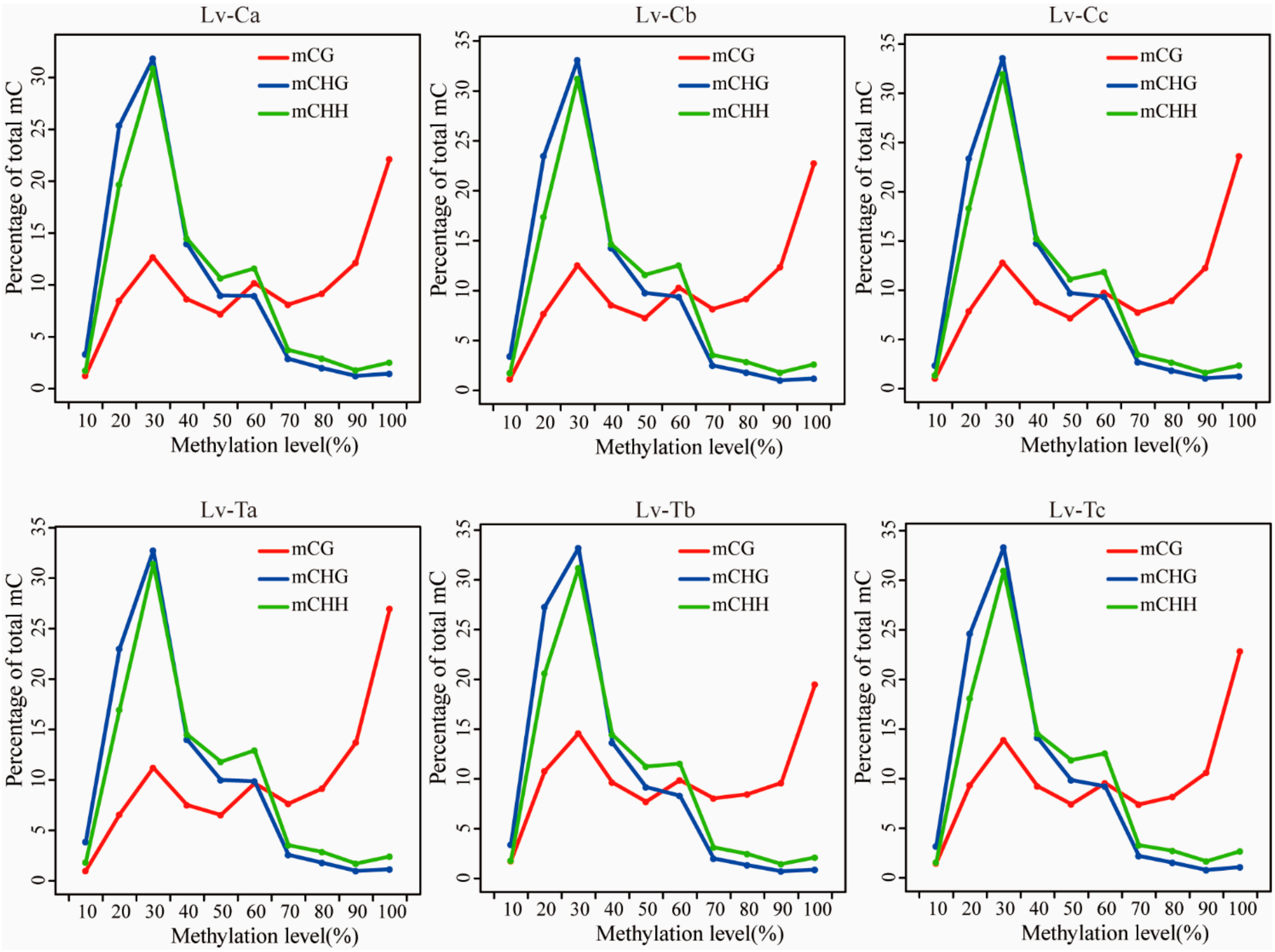

Supplementary Figure S4. Distribution plot of methylation levels for methylated Cs. The x-axis of the figure represents the level of methylation, ranging from 0% to 100%, with each category consisting of a 10% range. The y-axis represents the proportion of mCs at a specific level of methylation among all mCs.

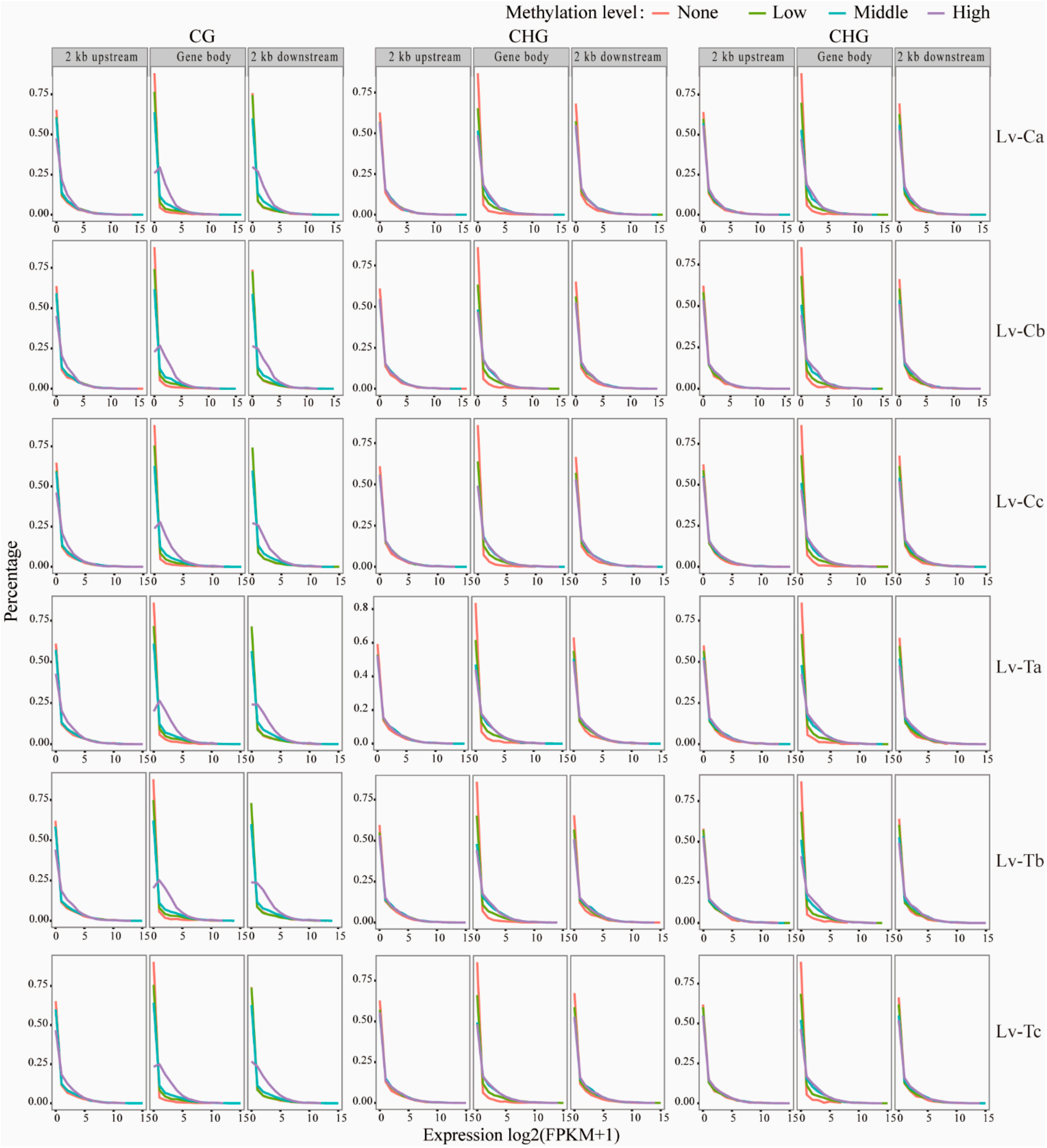

Supplementary Figure S5. The relationship between DNA methylation and gene expression. The expression profiles of methylated genes are compared with those of unmethylated genes. "None" indicates the set of non-methylated genes, and the level of methylation for methylated genes is divided into three groups: Low for genes with low methylation levels, Middle for genes with moderate methylation levels, and High for genes with high methylation levels. The x-axis represents the gene expression level (fpkm value +1, log transformed), and the y-axis represents the gene frequency. Different colors represent different levels of methylation, from left to right representing the upstream 2kbp region, gene region, and downstream 2kbp region.

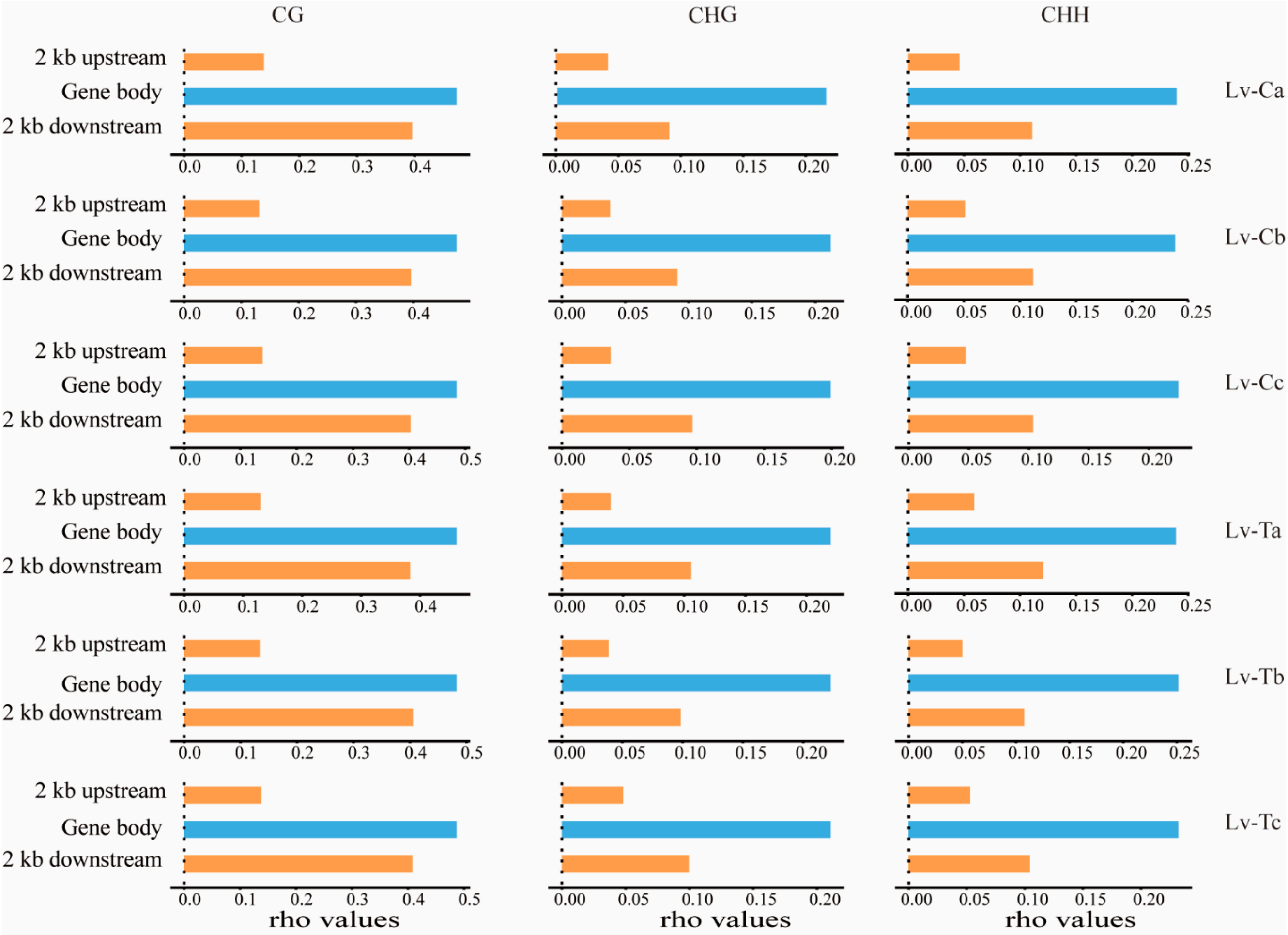

Supplementary Figure S6. Correlation between methylation and gene expression levels in the promoter, gene body, and 2 kb downstream regions. Rho > 0 represents a positive correlation, while rho < 0 represents a negative correlation. Upstream, gene body, and downstream represent the upstream, gene, and downstream regions of the gene, respectively.

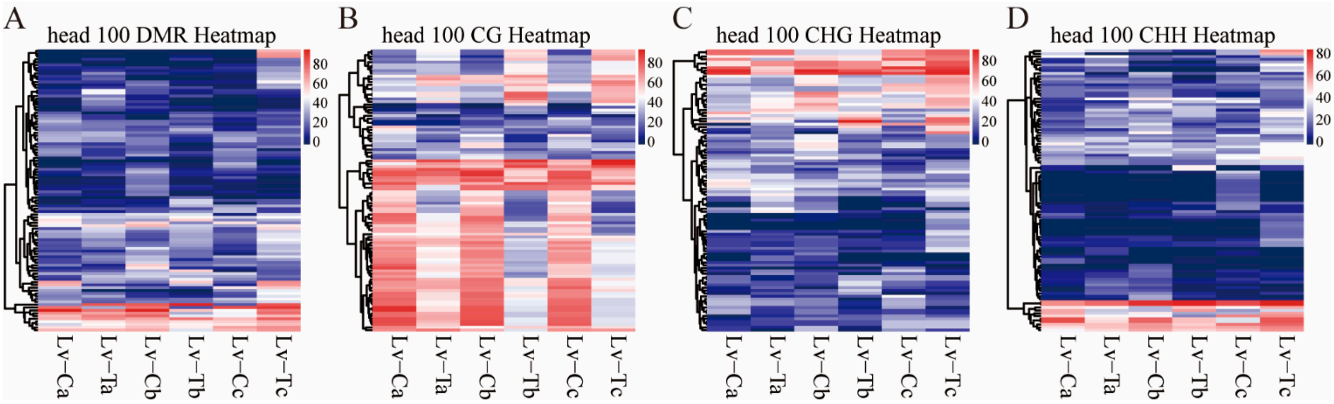

Supplementary Figure S7. Hheat maps of methylation levels within C, CG, CHG, and CHH differentially methylated regions (DMRs). Lv-T represents the cold-tolerant family, while Lv-C represents the normal family. Lv-Ta and Lv-Ca are the control groups under normal temperature at 28℃, Lv-Tb and Lv-Cb are the groups treated at 18℃, and Lv-Tc and Lv-Cc are the groups subjected to low-temperature stress at 10℃.

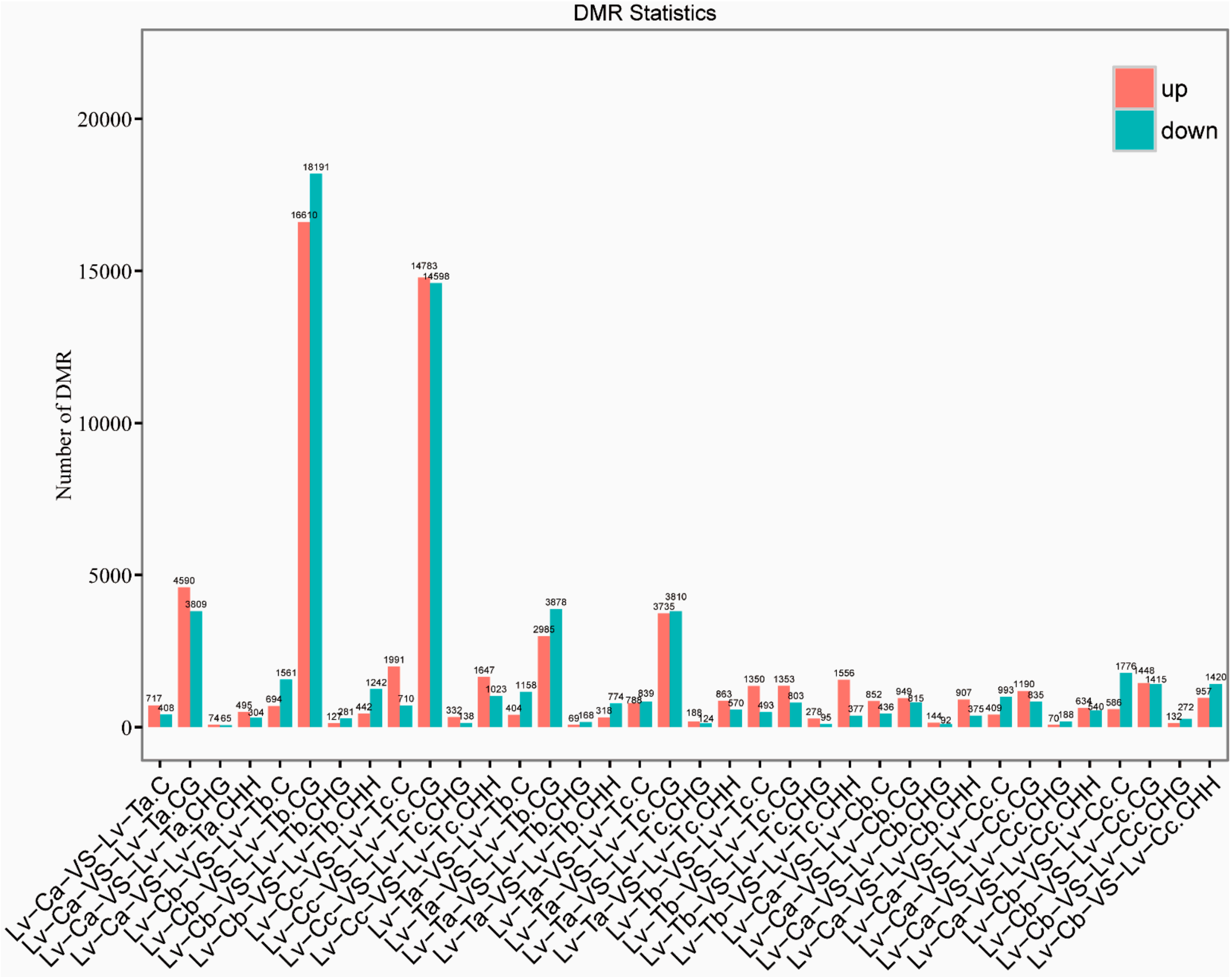

Supplementary Figure S8. DMR statistical plot. The x-axis represents the methylation patterns of each comparison group, and the y-axis represents the number of DMRs. The orange bars represent upregulation, while the green bars represent downregulation.

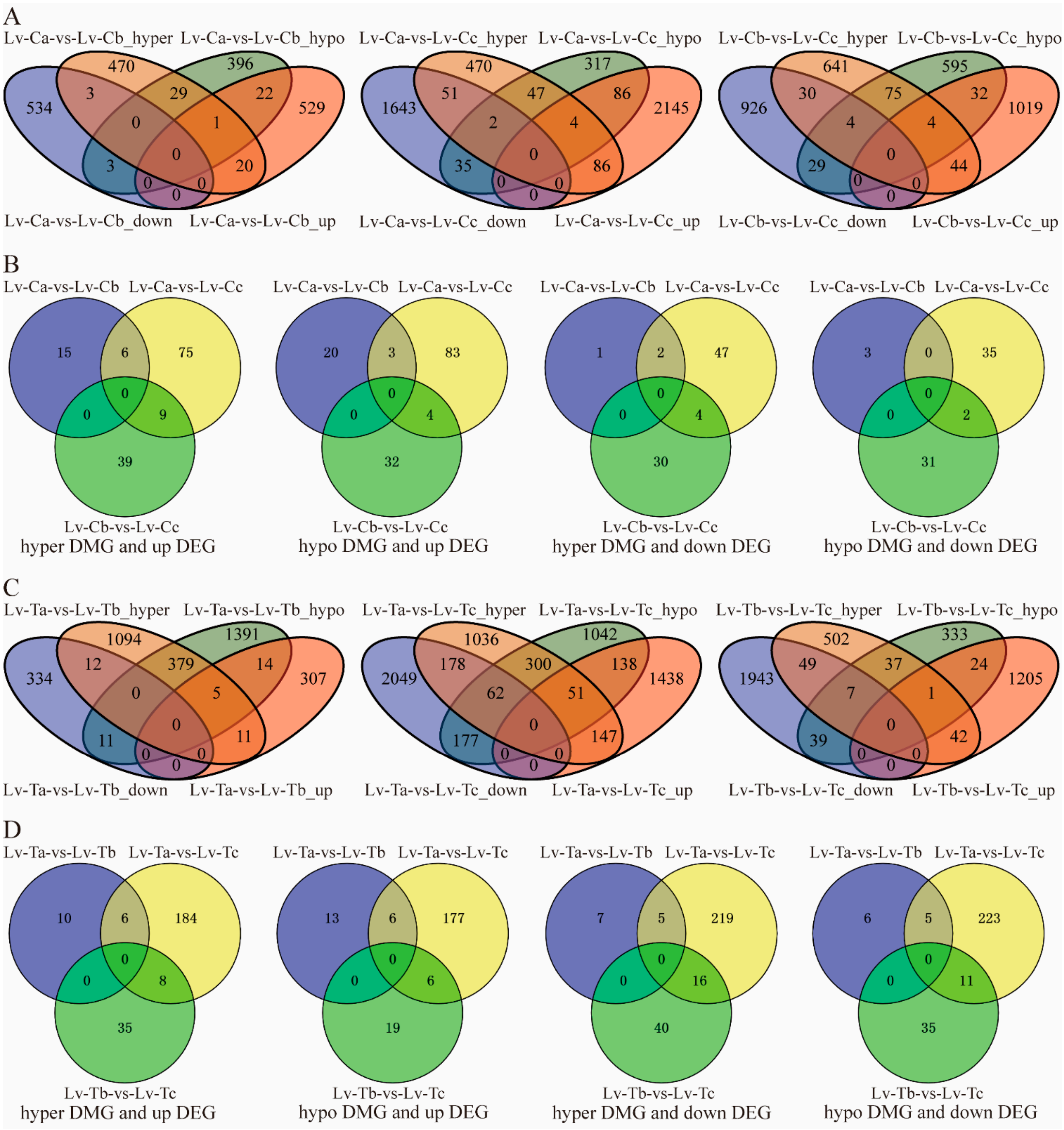

Supplementary Figure S9. Venn diagrams of differential methylated genes (DMRGs) with hypermethylation or hypomethylation and differentially expressed genes (DEGs) for each comparison group, represented by A, B, C, and D.

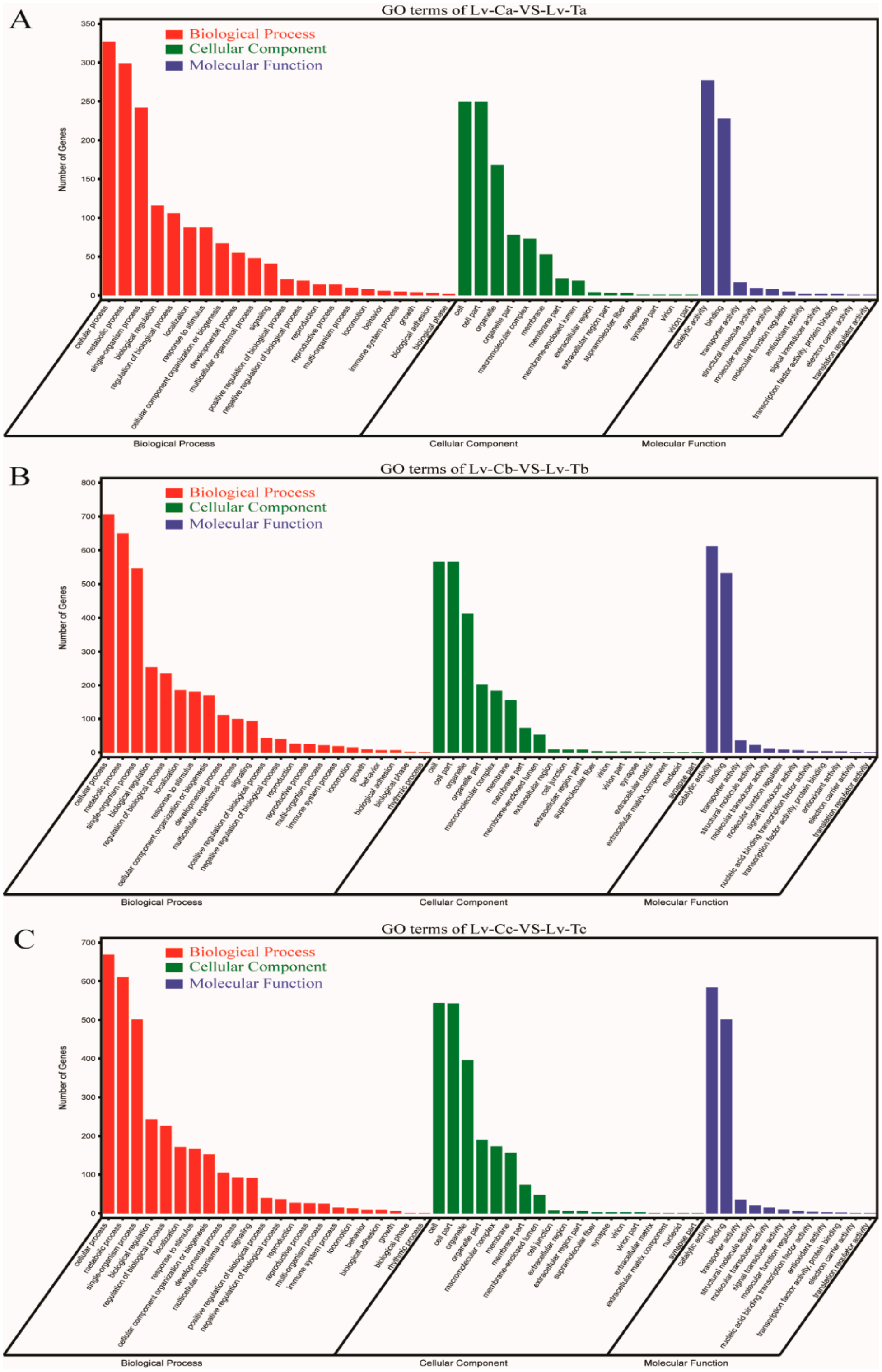

Supplementary Figure S10. GO enrichment analysis of DMRGs for each comparison group, represented by A, B, and C. The analysis was performed on differentially methylated region (DMR)-related genes in the comparison between cold-tolerant and normal families under low-temperature stress. The red bar represents Biological Process, the green bar represents Cellular Component, and the blue bar represents Molecular Function.
